# ﻿Three new microfungi (Ascomycota) species from southern China

**DOI:** 10.3897/mycokeys.111.136483

**Published:** 2024-12-11

**Authors:** Duhua Li, Mengyuan Zhang, Jinjia Zhang, Liguo Ma, Zhaoxue Zhang, Jie Zhang, Xiuguo Zhang, Jiwen Xia

**Affiliations:** 1 Shandong Provincial Key Laboratory for Biology of Vegetable Diseases and Insect Pests, College of Plant Protection, Shandong Agricultural University, Taian, 271018, China Shandong Agricultural University Taian China; 2 Institute of Plant Protection, Shandong Academy of Agricultural Sciences, Jinan, 250100, China Institute of Plant Protection, Shandong Academy of Agricultural Sciences Jinan China

**Keywords:** *Apiospora*, *Microdochium*, multigene phylogeny, *Pestalotiopsis*, Sordariomycetes taxonomy, three new taxa

## Abstract

*Apiospora*, *Microdochium* and *Pestalotiopsis* have been reported as plant pathogens, endophytes or saprotrophes worldwide. Combining multiple molecular markers with morphological characteristics, this study proposes three new species, viz. *Apiosporabambusigena*, *Microdochiumjianfenglingense* and *Pestalotiopsissolicola* from southern China. *Apiosporabambusigena* and *M.jianfenglingense* were collected from bamboo in Hainan Province and *P.solicola* was collected from soil in Yunnan Province. The morphologically similar and phylogenetically closely-related species were compared.

## ﻿Introduction

Bamboo, belonging to the subfamily Bambusoideae of the grass family Poaceae, is an evergreen plant with shallow roots, primarily distributed in the tropical and subtropical regions (Yeasmin et al. 2015; Dai et al. 2017). Bamboo is of high economic value; it can be used to build houses and furniture and has medicinal properties; bamboo shoots can be eaten (Jansen et al. 1995; Dransfield and Widjaja 1996; Lin 2004). Since the 18^th^ century, when Léveillé initiated the endeavour, research into bambusicolous fungi has been conducted (Léveillé 1845). Since entering the 21^st^ century, the diversity of bambusicolous fungi has been gradually explored (Hyde et al. 2002; Yeasmin et al. 2015; Dai et al. 2016, 2017).

Soil is an excellent culture media for the growth and development of various microorganisms, including fungi. Soil fungi play important roles in terrestrial ecosystems as decomposers in terrestrial ecosystems, participating in the carbon cycle and as pathogens and mutually beneficial symbiotic organisms of plants and animals (Taylor and Sinsabaugh 2015; Wu et al. 2024). Numerous soil-inhabiting fungi are specialised symbionts of forest trees or parasites on plant roots, but most are saprotrophs (Guarro et al. 2012).

Sordariomycetes was first introduced by Eriksson & Winka in 1997 and it was the second largest taxa in Ascomycota after Dothideomycetes, mainly characterised by non-lichenised, flask-shaped sporocarps (perithecia) and unitunicate asci (Eriksson and Winka 1997; Lumbsch 2000; Kirk et al. 2008; Hyde et al. 2013; Maharachchikumbura et al. 2015, 2016). Dai et al. (2017) reported many fungi in the taxa of Sordariomycetes on bamboo plants. Before that, reports on bambusicolous fungi were incomplete. During the experiment, various Sordariomycetes fungi were isolated from bamboo and soil, amongst which *Apiospora*, *Microdochium* and *Pestalotiopsis* accounted for a large proportion. *Apiospora* was first introduced by Saccardo in 1875 and is mainly characterised by globose to subglobose conidia, which are usually lenticular in side view, obovioid and pale-brown to brown (Hyde et al. 1998; Dai et al. 2017). *Microdochium* was first introduced by Sydow in 1924, mainly characterised by polyblastic, sympodial or annellidic conidiogenous cells with hyaline falcate conidia (Hernández-Restrepo et al. 2016). *Pestalotiopsis* was first introduced by Steyaert in 1949, mainly characterised by 5-celled conidia (Steyaert 1949). The classification and phylogenetic studies of bambusicolous fungi had important economic significance. Some bambusicolous fungi were pathogens that caused bamboo diseases, such as *Linearistromalineare* and *Calonectria* spp., which affected the growth and development of bamboo (Dai et al. 2017). On the other hand, some bambusicolous fungi were beneficial to humans. For example, the metabolite hypocrellin produced by *Shiraiabambusicola* is of great significance in anti-cancer treatment (Dai et al. 2017).

In this study, three new species of Sordariomycetes were found amongst samples collected in the Hainan and Yunnan Provinces of China. They were identified and classified by multi-locus analysis of tandem internal transcribed spacer (ITS), 28S large subunit ribosomal RNA gene (LSU), partial RNA polymerase II second-largest subunit (RPB2), translation elongation factor 1-alpha gene (TEF1α) and beta-tubulin gene region (TUB2) datasets. The new species are described and discussed, based on their morphological characteristics along with their molecular sequence data.

## ﻿Materials and methods

### ﻿Sampling site

Bambusoideae plant and soil specimens were collected from Hainan and Yunnan Provinces in China and important information was noted following Rathnayaka et al. (2024). Hainan Province (18°10′–20°10'N, 108°37′–111°03'E) is located in southern China, on the northern edge of the tropics, with an abundance of tropical climate resources and fertile soil. The climate is warm and humid, which is suitable for the growth of a variety of plants, especially bamboo. Thus, Hainan Province contains abundant resources of bambusicolous fungi. Yunnan Province (21°8′–29°15'N, 97°31′–116°11'E) of China is a mountain and plateau region on the country’s south-western frontier. Yunnan boasts the most diverse array of biological resources amongst all Chinese provinces, encompassing a wide range of plants and fungi originating from tropical, subtropical, temperate and alpine growth zones.

### ﻿Isolates and morphological analysis

For fresh plant tissues such as leaves, 6–8 sections (0.5 × 0.5 cm) of diseased or healthy tissues were selected and surface sterilised in 75% ethanol for 0.5 minutes, rinsed once in sterile distilled water and then immersed in a 5% sodium hypochlorite solution for 1 minute, followed by being rinsed thoroughly three times using sterile distilled water (Jiang et al. 2021a, 2021b). After rinsing three times in sterile distilled water, the tissue sections were transferred to sterilised and dried filter paper with sterilised tweezers and after the residual moisture dried, the tissue sections were spread on to potato dextrose agar (PDA: 200 g potato, 20 g dextrose, 15 g agar, sterilised distilled water added and filled to 1 litre, natural pH) medium plates and 2–4 sections were placed symmetrically on each PDA plate. For dried plant tissues such as withered twigs and other dry plant tissues, the tissues were observed under the body microscope and the single conidia were picked out with a slender picking needle and placed on to the PDA plates and 3–5 individual spores were picked out for each PDA plate. For soil samples, the dilution spreading method was adopted. A soil sample weighing 10 g was mixed with 90 ml of sterile distilled water in a conical flask. The flask was shaken at 200 rpm for 30 minutes and then allowed to settle briefly. The supernatant was extracted and diluted by 10, 100 and 1,000 times using a pipette. A volume of 100 μl of each diluted soil solution was dispensed on to PDA plates that contained streptomycin resistance materials (400 μl of 50 mg/ml streptomycin to 200 ml of PDA). A spreading rod was utilised to distribute the liquid evenly on the plates. Following a 10-minute stand, the culture plate was sealed. The prepared PDA plate was placed in a biological incubator at 25 °C for 3–4 days and then purified on a new medium plate after single colonies were grown to obtain a pure strain.

The individual colonies on the 7^th^ and 14^th^ days were morphologically observed and captured using a digital camera (Canon Powershot G7X). Additionally, the micromorphological characteristics of the colonies were examined with the aid of a stereomicroscope (Olympus SZX10) as well as a microscope (Olympus BX53). The two microscopes, equipped with Olympus DP80 and OPTIKA SC2000 HD colour digital cameras, observed the microscopic morphological characteristics of the structures generated during culture and captured and recorded the microscopic structure of the fungi. The pure cultured strains obtained in this experiment were cut into 0.5 × 0.5 cm pieces with a sterile scalpel and stored in a 2 ml frozen tube with 20% sterilised glycerine and 6–8 pieces were placed in each frozen tube and the frozen tube for fungal strain preservation was stored at -20 °C for further study (Wang et al. 2023; Zhang et al. 2023a).

Structural measurements were carried out using Digimizer software (v.5.6.0), with a minimum of 30 measurements for each characteristic, such as conidiophores, conidiogenous cells and conidia (Zhang et al. 2022a). The voucher specimens have been deposited in the Herbarium of the Department of Plant Pathology, Shandong Agricultural University, Taian, China (HSAUP) and the Herbarium Mycologicum Academiae Sinicae, Institute of Microbiology, Chinese Academy of Sciences, Beijing, China (HMAS). The ex-holotype living cultures have been archived in the Shandong Agricultural University Culture Collection (SAUCC) and the China General Microbiological Culture Collection Center (CGMCC). The taxonomic information of the new taxa has been submitted to MycoBank (http://www.mycobank.org, accessed on 29 Oct 2024).

### ﻿DNA extraction, PCR amplification and sequencing

The mycelium was scraped from the growing colonies on the medium plate and the mycelium tissue was processed into a fine powder by use of a mortar or mill. The DNA of the fungal genome was extracted through the utilisation of the modified cetyltrimethylammonium bromide (CTAB) method (Guo et al. 2000) or magnetic bead kit method (OGPLF-400, GeneOnBio Corporation, Changchun, China) (Zhang et al. 2023b). Table 1 lists the five genes viz. ITS, LSU, RPB2, TEF1α and TUB2 which were used in this paper, as well as the primers and PCR reaction procedures for locus amplification.

**Table 1.** Gene loci and corresponding PCR primers and programmes used in this study.

Locus	PCR primers	Sequence (5’ – 3’)	PCR cycles	References
ITS	ITS5	GGA AGT AAA AGT CGT AAC AAG G	(94 °C: 30 s, 55 °C: 30 s, 72 °C: 45 s) × 29 cycles	(White et al. 1990)
	ITS4	TCC TCC GCT TAT TGA TAT GC		
LSU	LR0R	GTA CCC GCT GAA CTT AAG C	(94 °C: 30 s, 48 °C: 50 s, 72 °C: 1 min 30 s) × 35 cycles	(Vilgalys and Hester 1990; Rehner and Samuels 1994)
	LR5	TCC TGA GGG AAA CTT CG		
RPB2	RPB2-5F2	GGG GWG AYC AGA AGA AGG C	(94 °C: 45 s, 60 °C: 45 s, 72 °C: 2 min) × 5 cycles, (94 °C: 45 s, 54 °C: 45 s, 72 °C: 2 min) × 30 cycles	(Liu et al. 1999; Sung et al. 2007)
	RPB2-7CR	CCC ATR GCT TGY TTR CCC AT		
TEF1α	EF1	ATG GGT AAG GAR GAC AAG AC	(95 °C: 30 s, 51 °C: 30 s, 72 °C: 1 min) × 35 cycles	(O’Donnell et al. 1998)
	EF2	GGA RGT ACC AGT SAT CAT GTT		
TUB2	Bt2a	GGT AAC CAA ATC GGT GCT GCT TTC	(95 °C: 30 s, 56 °C: 30 s, 72 °C: 1 min) × 35 cycles	(Glass and Donaldson 1995)
	Bt2b	ACC CTC AGT GTA GTG ACC CTT GGC		

The PCR reaction was conducted utilising an Eppendorf Master Thermocycler (Hamburg, Germany), and the detailed procedure for this reaction is provided in Table 1. Specifically, the PCR reaction was carried out in a 12 μl reaction system with the reaction composition of 6 μl 2 × Taq Master Mix (Dye Plus) (Vazyme, Nanjing, China, P112-01). The forward and reverse primers were 0.5 μl each (10 μM TsingKe, Qingdao, China), 1.5 μl template genomic DNA (about 10 ng/μl) and 3.5 μl sterilised distilled water. The resulting PCR products were examined by 1% agarose gel electrophoresis, stained with GelRed and the bands with the same size as the target fragment were observed under an ultraviolet lamp (Zhang et al. 2022b). Then the gel extraction kit (Cat: AE0101-C) (Shandong Sparkjade Biotechnology Co., Ltd.) was employed to recover the gel. The PCR amplified gene sequences were sequenced bidirectionally by Sangon Biotech Co., Ltd (Shanghai, China). Consistent sequences were obtained using MEGA v. 7.0 (Kumar et al. 2016). All sequences generated in this study have been deposited in GenBank, as detailed in Table 2.

**Table 2.** Names, strain numbers, substrates, regions and corresponding GenBank accession numbers of the taxa obtained in this study.

Species	Strain No.	Substrate	Region	GenBank Accession No.				
				ITS	LSU	RPB2	TEF1α	TUB2
*Apiosporabambusigena*	SAUCC 2446-2 ^T^	Bambusoideae sp. (leaf)	Jianfengling National Forest Park	PP702396	PP711785	–	PP716797	PP716801
	SAUCC 2446-6	Bambusoideae sp. (leaf)	Jianfengling National Forest Park	PP702397	PP711786	–	PP716798	PP716802
*Microdochiumjianfenglingense*	SAUCC 1862-2 ^T^	Bambusoideae sp. (leaf)	Jianfengling National Forest Park	PP702394	PP711783	PP716793	–	PP716799
	SAUCC 1862-5	Bambusoideae sp. (leaf)	Jianfengling National Forest Park	PP702395	PP711784	PP716794	–	PP716800
*Pestalotiopsissolicola*	SAUCC003804 ^T^	Soil	Kunming, Fumin County	OQ692020	–	–	OQ718737	OQ718795
	SAUCC003806	Soil	Kunming, Fumin County	OQ692021	–	–	OQ718738	OQ718796
	SAUCC003807	Soil	Kunming, Fumin County	OQ692022	–	–	OQ718739	OQ718797

Notes: Ex‐type strains are marked with “T”.

### ﻿Phylogenetic analyses

The nucleotide sequences of three new species were submitted to the NCBI’s GenBank nucleotide database (https://www.ncbi.nlm.nih.gov/, accessed on 29 Oct 2024) and the related species of all reference sequences were retrieved for phylogenetic analysis (Zhang et al. 2021). Employing the online MAFFT version 7 services and the automated policy (http://mafft.cbrc.jp/alignment/server/, accessed on 29 Oct 2024) to determine the arrangement of individual locus, multiple sequence analysis and, if necessary, manual correction (Katoh et al. 2019). The newly-generated sequence (Table 2) is compared with related sequences (Suppl. materials 5–7). To the species level, phylogenetic analysis was performed for each locus, followed by a combined multi-locus analysis.

The phylogenetic analyses relied on Maximum Likelihood (ML) and Bayesian Inference (BI) for the multi-locus studies. To determine the optimal evolutionary model for each segment in BI, MrModelTest v. 2.3 (Nylander 2004) was utilised and the selected models were integrated into the analysis framework. Both ML and BI were executed on the CIPRES Science Gateway portal (https://www.phylo.org/, accessed on 29 Oct 2024) (Miller et al. 2012), using RaxML-HPC2 on XSEDE v. 8.2.12 (Stamatakis 2014) for ML and MrBayes on XSEDE v. 3.2.7a (Huelsenbeck and Ronquist 2001; Ronquist and Huelsenbeck 2003; Ronquist et al. 2012) for BI. The default parameters were employed in the case of ML analyses, while BI was implemented with a rapid bootstrapping algorithm incorporating an automatic halt feature. The Bayesian analyses encompassed five concurrent runs spanning 5,000,000 generations, incorporating a stop rule and a sampling frequency of 50 generations. The burn-in fraction was set at 0.25 and posterior probabilities (PP) were calculated from the remaining trees. The resulting tree visualisations were generated using FigTree v. 1.4.4 (http://tree.bio.ed.ac.uk/software/figtree, accessed on 29 Oct 2024) or ITOL: Interactive Tree of Life (https://itol.embl.de/, accessed on 29 Oct 2024) (Letunic and Bork 2021) and the final layout of the trees was refined in Adobe Illustrator CC 2019.

## ﻿Results

### ﻿Phylogenetic analyses

During the extensive sample collection and identification process in Hainan and Yunnan, *Apiospora*, *Microdochium* and *Pestalotiopsis* fungi exhibited high isolation frequencies, occupying a significant proportion of the total isolated fungi. Consequently, this paper describes three novel *Apiospora*, *Microdochium* and *Pestalotiopsis* species.

#### ﻿*Apiosporabambusigena* sp. nov.

**Figure 1.**
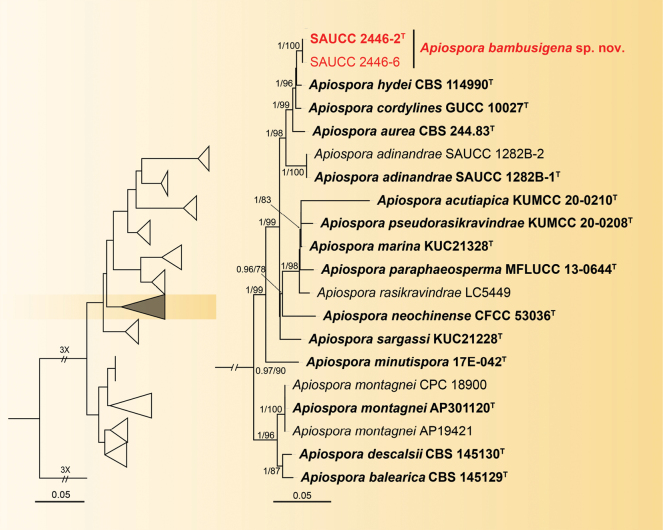
A Maximum Likelihood Inference tree based on a combined dataset of analysed ITS, LSU, TEF1α and TUB2 sequences. The Bayesian Inference Posterior Probability (left, BIPP ≥ 0.90) and the Maximum Likelihood Bootstrap Value (right, MLBV ≥ 75%) are shown as BIPP/MLBV above the nodes. Ex-type cultures are indicated in boldface and strains from the present study are in red. The scale bar at the bottom indicates 0.05 substitutions per site. To enhance the visual appeal of the evolutionary tree layout, certain branches are shortened by two diagonal lines (“//”) with the number of times. The figure shows partial branches of the evolutionary tree related to *Apiosporabambusigena* sp. nov. and the full evolutionary tree can be found in Suppl. material 1.

Phylogenetic analysis was conducted on 101 isolates with 100 isolates of *Apiospora* species designated as the ingroup and a single strain of *Arthriniumcaricicola* (CBS 145127) serving as the outgroup. The ultimate alignment encompassed 2140 concatenated characters, viz. 1–400 (ITS), 401–1200 (LSU), 1201–1600 (TEF1α) and 1601–2140 (TUB2). Amongst these, 1361 characters were constant, 234 were variable and parsimony-uninformative and 545 were parsimony-informative. The alignment comprises 918 distinct alignment patterns, with a percentage of gaps and fully undetermined characters standing at 24.62%. Estimated base frequencies were as follows: A = 0.235023, C = 0.243229, G = 0.260724, T = 0.261024; substitution rates AC = 1.392836, AG = 4.247629, AT = 1.251156, CG = 0.856792, CT = 5.000798 and GT = 1.000000; gamma distribution shape parameter α = 0.210667. Final ML Optimisation Likelihood: -17347.108598. The topology of the ML tree concurred with that derived from Bayesian Inference; thus, only the ML tree is presented. Based on the phylogeny of four genes, the 101 strains were categorised into 92 species (Suppl. material 1). The SYM+I+G model was proposed for ITS, the GTR+I+G for LSU and TUB2 and the HKY+G for TEF1α. MCMC analysis of these four tandem genes was performed over 3,535,000 generations in 70,702 trees. The initial 17,674 trees, representing the aging phase, were discarded, while the remaining trees contributed to calculating posterior probabilities in the majority rule consensus tree (Fig. 1; first value: BIPP ≥ 0.90 displayed). The alignment embodied 918 unique site patterns (ITS: 152, LSU: 182, TEF1α: 241, TUB2: 343).

#### ﻿*Microdochiumjianfenglingense* sp. nov.

**Figure 2.**
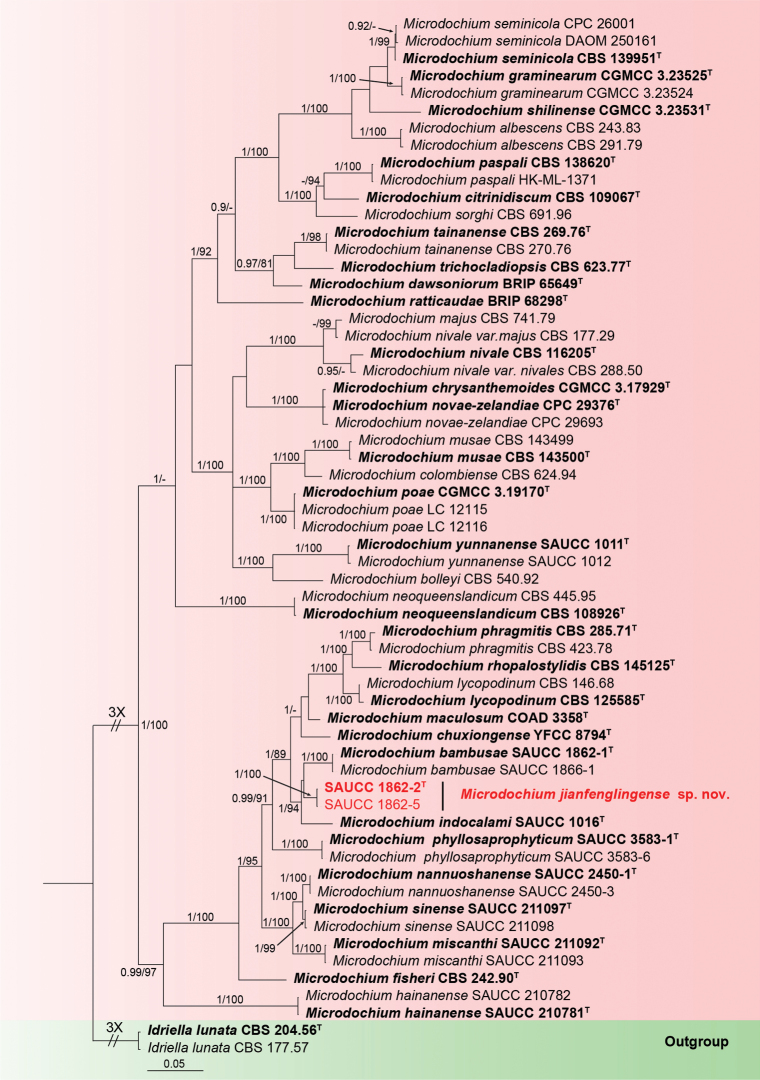
A Bayesian Inference tree based on a combined dataset of analysed ITS, LSU, RPB2 and TUB2 sequences. The Bayesian Inference Posterior Probability (left, BIPP ≥ 0.90) and the Maximum Likelihood Bootstrap Value (right, MLBV ≥ 75%) are shown as BIPP/MLBV above the nodes. Ex-type cultures are indicated in boldface, and strains from the present study are in red. The scale bar at the bottom indicates 0.05 substitutions per site. To enhance the visual appeal of the evolutionary tree layout, certain branches are shortened by two diagonal lines (“//”) with the number of times.

Phylogenetic analysis was conducted on 60 isolates comprising 58 ingroup isolates of *Microdochium* species and two outgroup strains of *Idriellalunata* (CBS 204.56, CBS 177.57). The final alignment encompassed 3034 concatenated characters, viz. 1–590 (ITS), 591–1423 (LSU), 1424–2244 (RPB2) and 2245–3034 (TUB2). Of these, 2228 were constant, 78 were variable and parsimony-uninformative and 728 were parsimony-informative. The alignment comprises 925 distinct alignment patterns, with a percentage of gaps and fully undetermined characters at 19.50%. Estimated base frequencies were as follows: A = 0.236313, C = 0.263048, G = 0.260231, T = 0.240408; substitution rates AC = 1.047671, AG = 5.296563, AT = 1.395107, CG = 0.980853, CT = 6.856348 and GT = 1.000000; gamma distribution shape parameter α = 0.125986. Final ML Optimisation Likelihood: -18130.357478. The topology of the ML tree concurred with that derived from Bayesian Inference; thus, only the Bayesian tree is presented. Based on the phylogeny of four genes, the 60 strains were categorised into 38 species (Fig. 2). The GTR+I+G model was proposed for ITS, LSU and TUB2 and the HKY+I+G for RPB2. MCMC analysis of these four tandem genes was performed over 1,885,000 generations in 56,552 trees. The initial 18,850 trees, representing the aging phase, were discarded, while the remaining trees contributed to calculating posterior probabilities in the majority rule consensus tree (Fig. 2; first value: BIPP ≥ 0.90 displayed). The alignment embodied 925 unique site patterns (ITS: 232, LSU: 92, RPB2: 336, TUB2: 265).

#### ﻿*Pestalotiopsissolicola* sp. nov.

**Figure 3.**
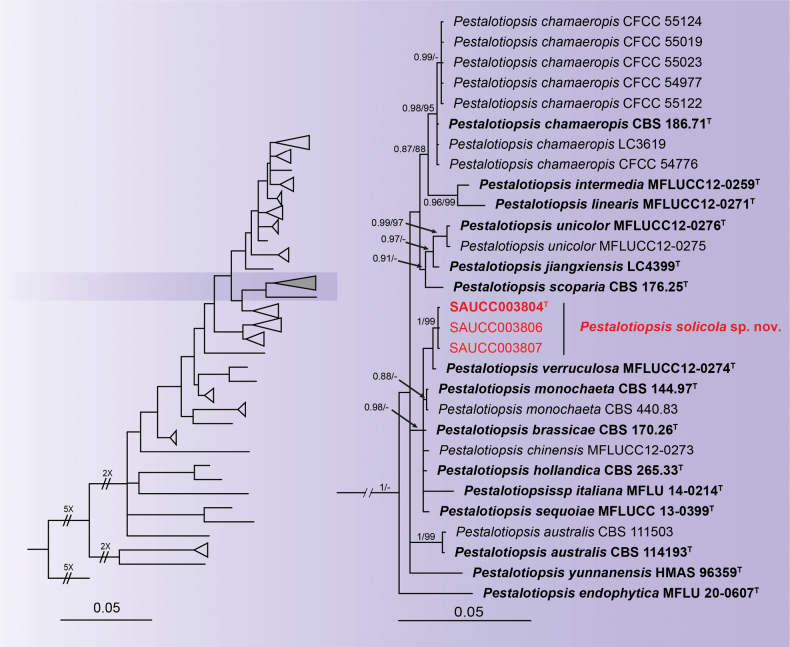
A Bayesian Inference tree based on a combined dataset of analysed ITS, TEF1α and TUB2 sequences. The Bayesian Inference Posterior Probability (left, BIPP ≥ 0.80) and the Maximum Likelihood Bootstrap Value (right, MLBV ≥ 75%) are shown as BIPP/MLBV above the nodes. Ex-type cultures are indicated in boldface and strains from the present study are in red. The scale bar at the bottom indicates 0.05 substitutions per site. To enhance the visual appeal of the evolutionary tree layout, certain branches are shortened by two diagonal lines (“//”) with the number of times. The figure shows partial branches of the evolutionary tree related to *Pestalotiopsissolicola* sp. nov. The full evolutionary tree can be found in Suppl. material 2.

Phylogenetic analysis was conducted on 184 isolates with 183 isolates of *Pestalotiopsis* species designated as the ingroup and a single strain of *Neopestalotiopsismagna* (MFLUCC 12-0652) serving as the outgroup. The ultimate alignment encompassed 1738 concatenated characters, viz. 1–538 (ITS), 539–884 (TEF1α) and 885–1738 (TUB2). Amongst these, 1017 characters were constant, 241 were variable and parsimony-uninformative and 480 were parsimony-informative. The alignment has 918 distinct alignment patterns. The proportion of gaps and fully undetermined characters stands at 24.01%. Estimated base frequencies were as follows: A = 0.233313, C = 0.301076, G = 0.212813, T = 0.252798; substitution rates AC = 0.948798, AG = 3.135932, AT = 1.068842, CG = 0.904564, CT = 4.060719 and GT = 1.000000; gamma distribution shape parameter α = 0.324087. Final ML Optimisation Likelihood: -13940.781313. The topology exhibited by the ML tree verifies the corresponding topology derived from Bayesian Inference; accordingly, only the Bayesian tree is displayed. Based on the phylogenetic analysis of three genes, the 184 strains were categorised into 109 species (Suppl. material 2). The HKY+I+G model was proposed for ITS, TEF1α and TUB2. MCMC analysis of these three tandem genes was performed over 3,325,000 generations in 49,878 trees. The initial 16,624 trees, which represent the aging phase of the analysis, are excluded, whereas the remaining trees are utilised for computing the posterior probability in the majority rule consensus tree (Fig. 3; first value: BIPP ≥ 0.80 displayed). The alignment embodied 918 unique site patterns (ITS: 177, TEF1α: 258, TUB2: 483).

In the phylogenetic analyses of *Apiospora*, 100 isolates are clustered as a monophyletic clade (Suppl. material 1). Isolates SAUCC 2446-2 and SAUCC 2446-6 formed a new clade sister to *Apiosporahydei* (CBS 114990) shown in the phylogram. Similarly, for *Microdochium*, 58 isolates are found as a monophyletic clade (Fig. 2). Isolates SAUCC 1862-2 and SAUCC 1862-5 formed a new clade sister to *Microdochiumbambusae* (SAUCC 1862-1, SAUCC 1866-1) shown in the phylogram. In the *Pestalotiopsis* phylogenetic analyses, 183 isolates are clustered as a monophyletic clade (Suppl. material 2). Isolates SAUCC003804, SAUCC003806 and SAUCC003807 formed a new clade sister to *Pestalotiopsisverruculosa* (MFLUCC12-0274) shown in the phylogram. The present research has identified three distinct new species: *Apiosporabambusigena*, *Microdochiumjianfenglingense* and *Pestalotiopsissolicola*.

### ﻿Taxonomy

#### ﻿ Apiospora bambusigena

Taxon classificationFungiXylarialesApiosporaceae

D.H. Li, Z.X. Zhang, J.W. Xia & X.G. Zhang sp. nov.

CA286D81-276A-5184-A182-C5BD78D419CF

853701

Fig. 4

##### Type.

China • Hainan Province: Jianfengling National Forest Park, on diseased leaves of Bambusoideae sp., 12 April 2023, D.H. Li (HMAS 352970, holotype), ex-holotype living culture SAUCC 2446-2 = CGMCC 3.27948.

##### Etymology.

The epithet *bambusigena* refers to the fungus produced on *bambusae*.

##### Description.

Conidiomata in culture sporodochial, aggregated or solitary, erumpent, black, surrounded by white mycelium. Conidiophores simple or confluent, hyaline, cylindrical to clavate, 7.8–18.8 × 3.7–4.6 μm, usually reduced to conidiogenous cells. Conidiogenous cells aggregative, hyaline, smooth, cylindrical, 5.2–8.8 × 3.0–4.6 μm. Conidia circular to slightly elliptical, immature conidia hyaline, rough, maturity conidia tanned to black, smooth, without a central scar, 15.0–18.0 × 14.5–17.0 μm, mean ± SD = 16.5 ± 1.0 × 16.0 ± 0.9 μm, n = 30. Sexual morph unknown.

**Figure 4.**
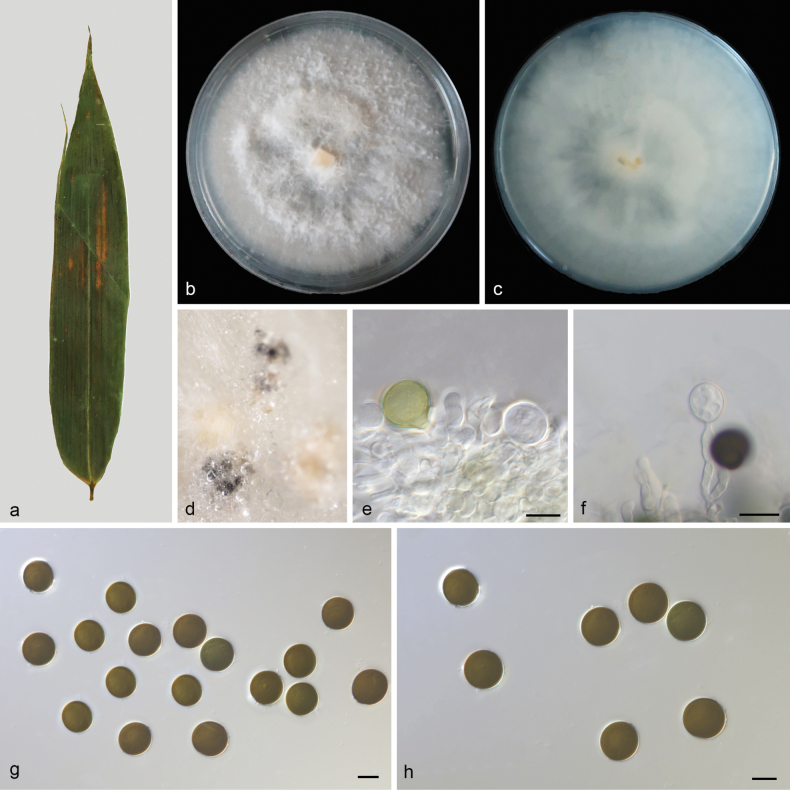
*Apiosporabambusigena* (HMAS 352970, holotype) **a** a leaf of Bambusoideae sp. **b, c** surface and reverse sides of colony after 14 days on PDA**d** colony overview with conidiomata **e, f** conidiogenous cells with conidia **g, h** conidia. Scale bars: 10 μm (**e–h**).

##### Culture characteristics.

The colonies diameter reached 80 mm after 14 days of dark culture at 25 °C on PDA, slightly rising above the surface of the substrate, non-uniform flocculent aerial mycelium and entire edge, white; reverse white.

##### Additional material studied.

China • Hainan Province: Jianfengling National Forest Park, on diseased leaves of Bambusoideae sp., 12 April 2023, D.H. Li, HSAUP 2446-6, living culture SAUCC 2446-6.

##### Notes.

Phylogenetic analyses of four combined sequences (ITS, LSU, TEF1α and TUB2) showed that *Apiosporabambusigena* constitutes a distinct clade, closely affiliated with *A.hydei* (CBS 114990). *Apiosporabambusigena* is distinguished from *A.hydei* by 13/598, 1/1152, 20/351 and 8/467 in ITS, LSU, TEF1α and TUB2 sequences, respectively. Morphologically, the conidia of *A.bambusigena* are narrower than *A.hydei* (15.0–18.0 × 14.5–17.0 μm vs. 15.0–17.0 × 19.0–22.0 μm) and the conidiophores of *A.bambusigena* are shorter than *A.hydei* (7.8–18.8 × 3.7–4.6 μm vs. 20–40 × 3–5 μm) (Crous and Groenewald 2013; Pintos and Alvarado 2021).

#### ﻿ Microdochium jianfenglingense

Taxon classificationFungiAmphisphaerialesAmphisphaeriaceae

D.H. Li, Z.X. Zhang, J.W. Xia & X.G. Zhang sp. nov.

1FEC9B14-485C-5143-8A02-F4A169E61AB0

853702

Fig. 5

##### Type.

China • Hainan Province: Jianfengling National Forest Park, on diseased leaves of Bambusoideae sp., 12 April 2023, D.H. Li (HMAS 352971, holotype), ex-holotype living culture SAUCC 1862-2 = CGMCC 3.27947.

##### Etymology.

The epithet *jianfenglingense* refers to the Jianfengling National Forest Park, where the holotype was collected.

##### Description.

Conidiophores simple, hyaline, cylindrical to clavate, sometimes reduced to conidiogenous cells. Conidiogenous cells straight or slightly curved, 15.0–25.5 × 1.9–3.0 μm, monoblastic or polyblastic, terminal, denticulate, transparent, smooth, cylindrical and septate and produced on aerial mycelia. Conidia are solitary, hyaline, often 3-septate, spindle, oblong to ellipsoid, straight or curved, 13.0–24.0 × 2.5–4.5 μm, mean ± SD = 17.5 ± 2.5 × 3.4 ± 0.5 μm, n = 30, multi-guttulate and sometimes borne directly from the hyphae. No chlamydospores were observed. Sexual morph unknown.

**Figure 5.**
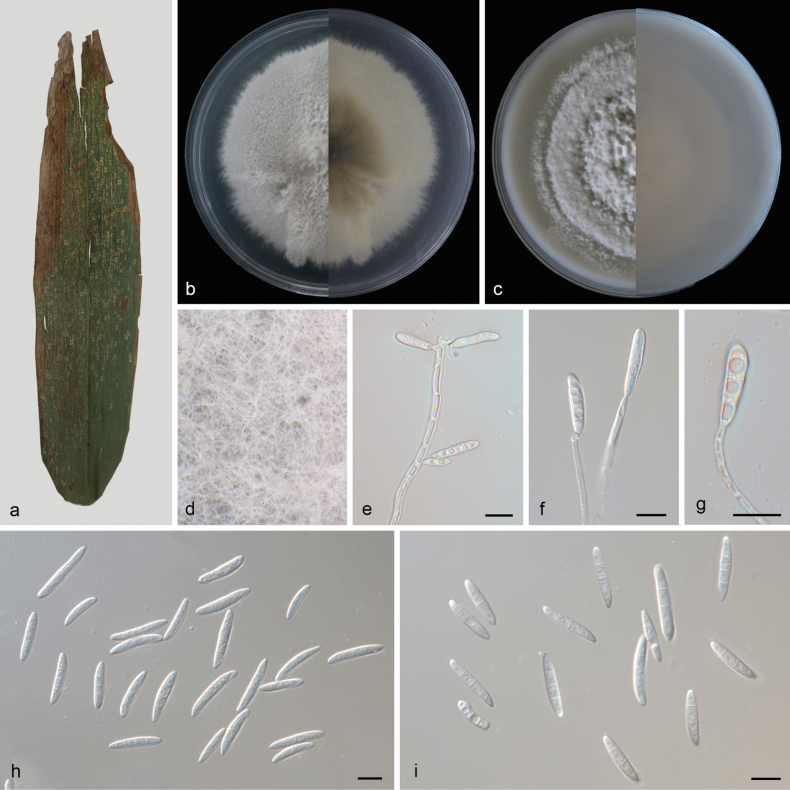
*Microdochiumjianfenglingense* (HMAS 352971, holotype) **a** a leaf of Bambusoideae sp. **b, c** surface and reverse sides of the colony after 14 days on PDA, OA **d** colony overview **e–g** conidiogenous cells with conidia **h, i** conidia. Scale bars: 10 μm (**e–i**).

##### Culture characteristics.

The colonies diameter reached 69–72 mm after 14 days of dark culture at 25 °C on PDA, colonies exhibited concentric spreading, fluffy, marginal aerial mycelium white to cream, gradually turning tawny towards the centre; reverse white to tawny. The colonies diameter reached 64–74 mm after 14 days of dark culture at 25 °C on OA, colonies concentrically spreading, fluffy, aerial mycelium milky white, substrate mycelium grey in the medium; reverse white.

##### Additional material studied.

China • Hainan Province: Jianfengling National Forest Park, on diseased leaves of Bambusoideae sp., 12 April 2023, D.H. Li, HSAUP 1862-5, living culture SAUCC 1862-5.

##### Notes.

Phylogenetic analyses of four combined sequences (ITS, LSU, RPB2 and TUB2) showed that *Microdochiumjianfenglingense* constitutes a distinct clade, closely affiliated with *M.bambusae* (SAUCC 1862-1 and SAUCC 1866-1) and *M.indocalami* (SAUCC 1016). *Microdochiumjianfenglingense* is distinguished from *M.bambusae* (SAUCC 1866-1) by 7/535, 3/828 and 59/912 characters and from *M.indocalami* (SAUCC 1016) by 24/539, 1/832 and 48/840 characters in ITS, LSU and RPB2 sequences, respectively. Morphologically, the conidia of *M.jianfenglingense* are longer than *M.bambusae* and *M.indocalami* (13.0–24.0 × 2.5–4.5 μm vs. 13.0–17.0 × 2.5–3.5 μm vs. 13.0–15.5 × 3.5–5.5 μm). Conidiogenous cells of *M.jianfenglingense* are shorter than *M.bambusae* and *M.indocalami* (15.0–25.5 × 1.9–3.0 μm vs. 17.4–30.0 × 2.5–3.0 μm vs. 11.0–28.3 × 1.5–2.9 μm) (Huang et al. 2020; Zhang et al. 2023).

#### ﻿ Pestalotiopsis solicola

Taxon classificationFungiAmphisphaerialesPestalotiopsidaceae

D.H. Li, Z.X. Zhang, J.W. Xia & X.G. Zhang sp. nov.

ECBD5DDF-ED18-5511-BD49-A8A73F0F5A8B

854062

Fig. 6

##### Type.

China • Yunnan Province, Kunming, Fumin County, in soil, 20 May 2023, D.H. Li (HMAS 352972, holotype), ex-holotype living culture SAUCC 003804 = CGMCC 3.22681.

##### Etymology.

The epithet refers to the substrate “soil” from which the holotype was isolated.

##### Description.

Conidiomata appear as sporodochial structures in culture, solitary or aggregated, black, erumpent, exuding dark conidial masses. Conidiophores simple or confluent, hyaline, cylindrical to clavate, usually reduced to conidiogenous cells. Conidiogenous cells aggregative, smooth, cylindrical to clavate, hyaline, 15.0–40.4 × 2.7–7.2 μm. Conidia fusoid, straight or slightly curved, 4-septate, smooth, slightly constricted at the septa, 24.3–32.4 × 8.0–10.0 μm; basal cell obconic with a truncate base, 2.0–5.9 μm long, thin-walled, hyaline, basal appendages single, unbranched, tubular, straight or slightly bent, 10.3–13.4 μm long; median cells 3, trapezoid or subcylindrical, thick-walled, pale brown to brown, 18.8–21.3 μm long, specifically, the first median cell from base 3.1–7.5 μm long, the second median cell 6.2–8.2 μm long, the third median cell 4.8–6.9 μm long; apical cell conic with an acute apex, hyaline, thin-walled, 2.1–5.5 μm long; apical appendages 2–5, unbranched, tubular, straight or slightly curved, 25.0–32.0 μm long. Sexual morph unknown.

**Figure 6.**
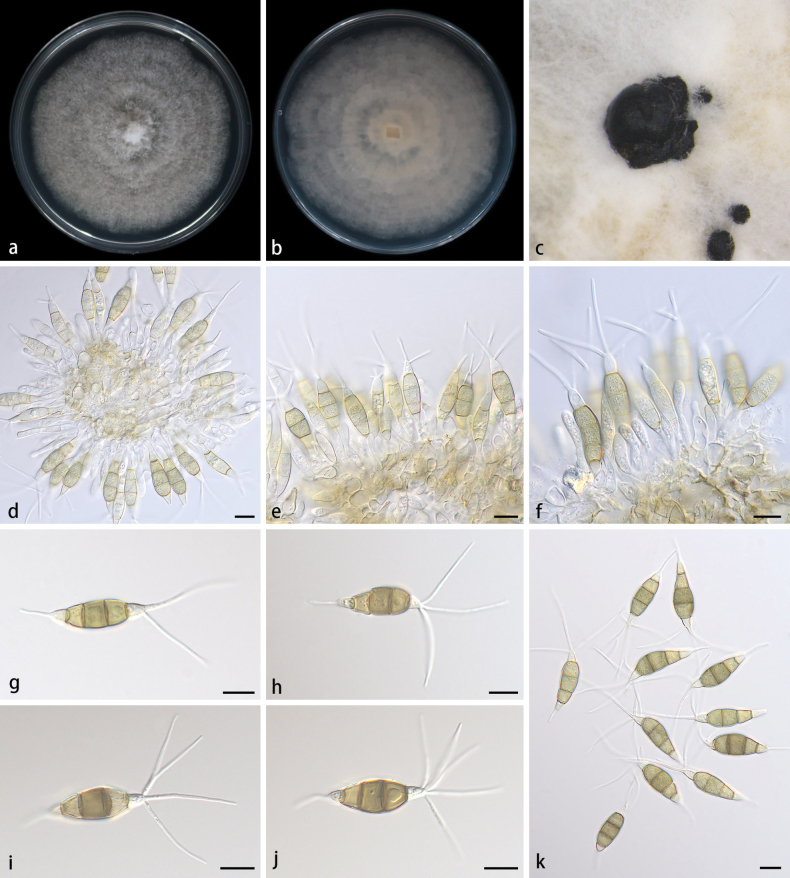
*Pestalotiopsissolicola* (HMAS 352972, holotype) **a, b** surface and reverse sides of colony after 7 days on PDA**c** colony overview with conidiomata **d–f** conidiogenous cells with conidia **g–k** conidia. Scale bars: 10 μm (**d–k**).

##### Culture characteristics.

The colonies diameter reached 75–80 mm after 7 days of dark culture at 25 °C on PDA, whitish, flat, with flocculent aerial mycelium forming concentric rings and entire edge; reverse white.

##### Additional material studied.

China • Yunnan Province, Kunming, Fumin County, in soil, 20 May 2023, D.H. Li, HSAUP 003806, living culture SAUCC 003806; ibid., HSAUP 003807, living culture SAUCC 003807.

##### Notes.

Phylogenetic analyses of three combined sequence (ITS, TEF1α and TUB2) showed that *Pestalotiopsissolicola* was found to constitute a distinct clade, closely affiliated with *P.brassicae* (CBS 170.26), *P.chinensis* (MFLUCC 12-0273), *P.hollandica* (CBS 265.33), *P.italiana* (MFLU 14-0214), *P.monochaeta* (CBS 144.97 and CBS 440.83), *P.sequoiae* (MFLUCC 13-0399) and *P.verruculosa* (MFLUCC 12-0274). *P.solicola* differs from: *P.brassicae* by 6/261 bp in TEF1α, *P.hollandica* by 6/273 bp in TEF1α and 6/769 bp in TUB2, *P.italiana* by 9/442 bp in ITS, 7/266 bp in TEF1α and 3/446 bp in TUB2, *P.monochaeta* by 15/282 bp in TEF1α, *P.verruculosa* by 1/540 bp in ITS and 2/273 bp in TEF1α. In addition, a small phylogenetic tree containing the individual genes TEF1α and TUB2 of these species was added (Suppl. materials 3, 4). In morphology, *P.solicola* is closely related to seven other species, but there are also differences. For more details, see the morphological comparison of the species in Table 3. The differences between *P.solicola* and other species mainly focus on the number of apical appendages, the size of conidia and the culture characteristics of the PDA medium. (Maharachchikumbura et al. 2012, 2014; Liu et al. 2015; Hyde et al. 2016).

**Table 3.** Morphological comparison between *P.solicola* and other closely-related species.

Species		*P.brassicae*	*P.hollandica*	*P.monochaeta*	*P.chinensis*	*P.verruculosa*	*P.italiana*	*P.sequoiae*	*P.solicola*
**Culture characteristics**		whitish	whitish to pale grey	whitish to pale yellow	whitish to pale yellow, reverse yellow to pale orange	whitish to pale yellow, reverse yellow to pale orange	whitish to pale grey	whitish	whitish
**Conidiomata**		dark brown to black	dark brown to black	dark brown to black	black	black	dark brown to black	black	black
**Conidia**		29–40 × 8–11.5 μm	25–34 × 8.5–10.5 μm	25–42 × 7–11.5 μm	23–32 × 7–9 μm	28–35 × 9–11 μm	26–35 × 8–11 μm	21–30 × 7.5–10 μm	24.3–32.4 × 8–10 μm
**Basal cell**		5–8.5 μm	5–7.5 μm	5.5–9.5 μm	5–7 μm	5–7 μm	5–7 μm	2.9–5.7 μm	2–5.9 μm
**Median cells**	**shape**	doliiform to subcylindrical	doliiform, verruculose	doliiform, verruculose	doliiform to cylindrical	doliiform to cylindrical, with thick verruculose walls	doliform to cylindrical, with thick verruculose walls	cylindrical	trapezoid or subcylindrical
	**size**	20–25 μm	16.5–24 μm	17–26 μm	20–22 μm	18–26 μm	18–28 μm	14.7–20 μm	18.8–21.3 μm
	**colour**	brown to olivaceous	concolourous	concolourous	concolorous, olivaceous	concolorous, olivaceous	concolorous, olivaceous	pale brown to brown and concolourous	pale brown to brown
	**second cell**	5.5–9 μm	5–8.5 μm	5–8.5 μm	6–7 μm	6–9 μm	5.5–8.5 μm	4.1–7 μm	3.1–7.5 μm
	**third cell**	7–9.5 μm	6–9 μm	7–9 μm	7–7.5 μm	6–9 μm	6–9 μm	5.4–6.9 μm	6.2–8.2 μm
	**fourth cell**	6–9 μm	6–8 μm	7–9 μm	6–7.5 μm	6–9 μm	6–9 μm	4.6–6.7 μm	4.8–6.9 μm
**Apical cell**	**shape**	cylindrical to subcylindrical	conical	conical	conical to subcylindrical	conical to subcylindrical	conical to subcylindrical	conical	conical with an acute apex
	**size**	3.5–7 μm	3.5–5 μm	4–6.5 μm	3–6 μm	4–6 μm	4–6.5 μm	2.9–4.8 μm	2.1–5.5 μm
**Apical appendages**	**quantity**	3–5 (mostly 4)	1–4	1	1–3 (mostly 3)	2–6 (mostly 3–4)	2–5 (mostly 3–4)	mostly 4	2–5
	**size**	27–50 μm	20–40 μm	40–75 μm	25–30 μm	25–40 μm	20–40 μm	3–17 μm	25–32 μm
**Basal appendage**	**size**	10–25 μm	3–9 μm	6–14 μm	7–11 μm	8–12 μm	6–10 μm	4–11 μm	10.3–13.4 μm

## ﻿Discussion

*Apiospora* was introduced by Saccardo, with *A.montagnei* Sacc. as the type species (Saccardo 1875). Characterised by multi-locular perithecial stromata enclosing hyaline ascospores that are encompassed by a thick gelatinous sheath, the sexual morphs of *Apiospora* are distinct (Dai et al. 2017; Pintos and Alvarado 2021). Meanwhile, the asexual morphs of *Apiospora* are identified by their basauxic conidiogenesis and globose to subglobose conidia, which typically appear lenticular or obovoid in side view and range in colour from pale brown to brown (Kunze 1817; Hyde et al. 1998). *Apiospora* is similar to *Arthrinium* and *Neoarthrinium* in morphology, especially the basauxic conidiogenesis (Jiang et al. 2022; Liu et al. 2023). Most species of *Apiospora*, *Arthrinium* and *Neoarthrinium* are quite similar to each other in morphology; thus, it is difficult to distinguish them without molecular phylogenetic data.

*Microdochium* was established by Sydow with *M.phragmitis* as the type species (Sydow 1924). The sexual morphs of *Microdochium* are characterised by perithecial stromata with oblong to clavate asci that produce fusiform or oblong, hyaline ascospores. The asexual morphs of *Microdochium* are characterised by monoblastic or polyblastic conidiogenous cells and hyaline falcate conidia (Hernández-Restrepo et al. 2016; Liu et al. 2022; Zhang et al. 2023). *Microdochium* is similar to *Idriella* in morphology; however, they can be separated by the pigmentation of their conidiogenous cells (Hernández-Restrepo et al. 2016).

Based on the conidial forms, Steyaert (1949) split *Pestalotia* into three genera, namely *Pestalotia*, *Pestalotiopsis* and *Truncatella*. Specifically, the genus *Pestalotia* was introduced for species with 6-celled conidia, *Pestalotiopsis* for species with 5-celled conidia and *Truncatella* for species with 4-celled conidia. The introduction of the genus *Pestalotiopsis* by Steyaert (1949) to accommodate the 5-celled conidial forms of *Pestalotia* resulted in appreciable controversy from Moreau and Guba (Moreau 1949; Steyaert 1949; Guba 1956, 1961). *Pestalotiopsis* species are morphologically diverse in conidial morphology and phylogenetic analyses of different gene regions have established that *Pestalotiopsis* comprises three distinct lineages (Jeewon et al. 2003; Maharachchikumbura et al. 2011, 2012). Based on these findings, Maharachchikumbura et al. (2014) divided *Pestalotiopsis* into three genera: *Pestalotiopsis*, *Neopestalotiopsis* and *Pseudopestalotiopsis*. Phenotypic analyses of conidial characters coupled with phylogenetic analyses of sequence data were used to clarify species boundaries in the three genera (Maharachchikumbura et al. 2014).

In this study, we collected parasitic or saprotrophic fungi on Bambusoideae plant or soil specimens from terrestrial habitats in Hainan and Yunnan Province, China. Based on morphological characteristics and phylogenetic data, *Apiosporabambusigena* sp. nov., *Microdochiumjianfenglingense* sp. nov. and *Pestalotiopsissolicola* sp. nov. are introduced.

## Supplementary Material

XML Treatment for Apiospora bambusigena

XML Treatment for Microdochium jianfenglingense

XML Treatment for Pestalotiopsis solicola

## ﻿Additional information

### Author ORCIDs

Duhua Li https://orcid.org/0009-0006-5200-2034

Zhaoxue Zhang https://orcid.org/0000-0002-4824-9716

Xiuguo Zhang https://orcid.org/0000-0001-9733-8494

Jiwen Xia https://orcid.org/0000-0002-7436-7249

### Data availability

All of the data that support the findings of this study are available in the main text or Supplementary Information.

## Supplementary materials

Supplementary material 1 A Maximum Likelihood Inference tree based on a combined dataset of analysed ITS, LSU, TEF1α and TUB2 sequences Data type docx Explanation note The Bayesian Inference Posterior Probability (left, BIPP ≥ 0.90) and the Maximum Likelihood Bootstrap Value (right, MLBV ≥ 75%) are shown as BIPP/MLBV above the nodes. Ex-type cultures are indicated in boldface and strains from the present study are in red. The tree was rooted in *Arthriniumcaricicola* (CBS 145127). The scale bar at the bottom indicates 0.05 substitutions per site. In order to make the layout of the evolutionary tree beautiful, some branches are shortened by two diagonal lines (“//”) with the number of times. https://binary.pensoft.net/file/1196748This dataset is made available under the Open Database License (http://opendatacommons.org/licenses/odbl/1.0/). The Open Database License (ODbL) is a license agreement intended to allow users to freely share, modify, and use this Dataset while maintaining this same freedom for others, provided that the original source and author(s) are credited.Duhua Li, Mengyuan Zhang, Jinjia Zhang, Liguo Ma, Zhaoxue Zhang, Jie Zhang, Xiuguo Zhang, Jiwen Xia

Supplementary material 2 A Bayesian Inference tree based on a combined dataset of analysed ITS, TEF1α and TUB2 sequences Data type docx Explanation note The Bayesian Inference Posterior Probability (left, BIPP ≥ 0.80) and the Maximum Likelihood Bootstrap Value (right, MLBV ≥ 75%) are shown as BIPP/MLBV above the nodes. Ex-type cultures are indicated in boldface and strains from the present study are in red. The tree was rooted in *Neopestalotiopsismagna* (MFLUCC 12-0652). The scale bar at the bottom indicates 0.05 substitutions per site. In order to make the layout of the evolutionary tree beautiful, some branches are shortened by two diagonal lines (“//”) with the number of times. https://binary.pensoft.net/file/1196749This dataset is made available under the Open Database License (http://opendatacommons.org/licenses/odbl/1.0/). The Open Database License (ODbL) is a license agreement intended to allow users to freely share, modify, and use this Dataset while maintaining this same freedom for others, provided that the original source and author(s) are credited.Duhua Li, Mengyuan Zhang, Jinjia Zhang, Liguo Ma, Zhaoxue Zhang, Jie Zhang, Xiuguo Zhang, Jiwen Xia

Supplementary material 3 Phylogenetic tree of *Pestalotiopsissolicola* and related species based on TEF1α Data type docx https://binary.pensoft.net/file/1196750This dataset is made available under the Open Database License (http://opendatacommons.org/licenses/odbl/1.0/). The Open Database License (ODbL) is a license agreement intended to allow users to freely share, modify, and use this Dataset while maintaining this same freedom for others, provided that the original source and author(s) are credited.Duhua Li, Mengyuan Zhang, Jinjia Zhang, Liguo Ma, Zhaoxue Zhang, Jie Zhang, Xiuguo Zhang, Jiwen Xia

Supplementary material 4 Phylogenetic tree of *Pestalotiopsissolicola* and related species based on TUB2 Data type docx https://binary.pensoft.net/file/1196751This dataset is made available under the Open Database License (http://opendatacommons.org/licenses/odbl/1.0/). The Open Database License (ODbL) is a license agreement intended to allow users to freely share, modify, and use this Dataset while maintaining this same freedom for others, provided that the original source and author(s) are credited.Duhua Li, Mengyuan Zhang, Jinjia Zhang, Liguo Ma, Zhaoxue Zhang, Jie Zhang, Xiuguo Zhang, Jiwen Xia

Supplementary material 5 GenBank accession numbers of the taxa used in *Apiospora* phylogenetic reconstruction Data type docx https://binary.pensoft.net/file/1196752This dataset is made available under the Open Database License (http://opendatacommons.org/licenses/odbl/1.0/). The Open Database License (ODbL) is a license agreement intended to allow users to freely share, modify, and use this Dataset while maintaining this same freedom for others, provided that the original source and author(s) are credited.Duhua Li, Mengyuan Zhang, Jinjia Zhang, Liguo Ma, Zhaoxue Zhang, Jie Zhang, Xiuguo Zhang, Jiwen Xia

Supplementary material 6 GenBank accession numbers of the taxa used in *Microdochium* phylogenetic reconstruction Data type docx https://binary.pensoft.net/file/1196753This dataset is made available under the Open Database License (http://opendatacommons.org/licenses/odbl/1.0/). The Open Database License (ODbL) is a license agreement intended to allow users to freely share, modify, and use this Dataset while maintaining this same freedom for others, provided that the original source and author(s) are credited.Duhua Li, Mengyuan Zhang, Jinjia Zhang, Liguo Ma, Zhaoxue Zhang, Jie Zhang, Xiuguo Zhang, Jiwen Xia

Supplementary material 7 GenBank accession numbers of the taxa used in *Pestalotiopsis* phylogenetic reconstruction Data type docx https://binary.pensoft.net/file/1196754This dataset is made available under the Open Database License (http://opendatacommons.org/licenses/odbl/1.0/). The Open Database License (ODbL) is a license agreement intended to allow users to freely share, modify, and use this Dataset while maintaining this same freedom for others, provided that the original source and author(s) are credited.Duhua Li, Mengyuan Zhang, Jinjia Zhang, Liguo Ma, Zhaoxue Zhang, Jie Zhang, Xiuguo Zhang, Jiwen Xia
