# Supplementary material for: ﻿Three new microfungi (Ascomycota) species from southern China
Source: MycoKeys. 2024 Dec 11;111:87–110. doi: 10.3897/mycokeys.111.136483 (PMC11656163; doi:10.3897/mycokeys.111.136483)
Supplement: Supplementary material 2 — A Bayesian Inference tree based on a combined dataset of analysed ITS, TEF1α and TUB2 sequences [file mycokeys-111-087-s002.docx]

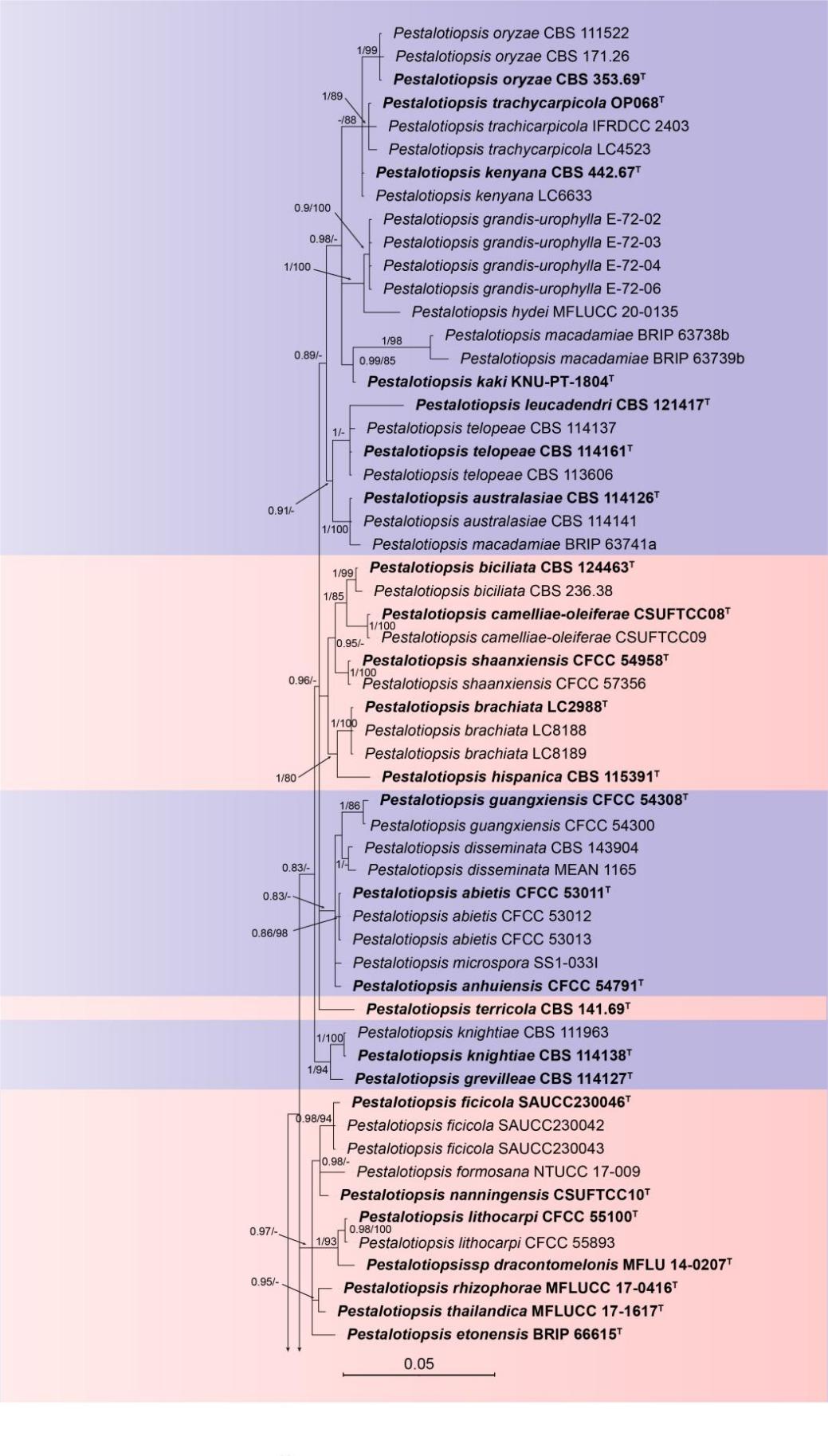


**Figure S2.** A Bayesian Inference tree based on a combined dataset of analysed ITS, TEF1α and TUB2 sequences. The Bayesian Inference Posterior Probability (left, BIPP ≥ 0.80) and the Maximum Likelihood Bootstrap Value (right, MLBV ≥ 75%) are shown as BIPP/MLBV above the nodes. Ex-type cultures are indicated in boldface and strains from the present study are in red. The tree was rooted in *Neopestalotiopsis magna* (MFLUCC 12-0652). The scale bar at the bottom indicates 0.05 substitutions per site. In order to make the layout of the evolutionary tree beautiful, some branches are shortened by two diagonal lines (“//”) with the number of times.

**
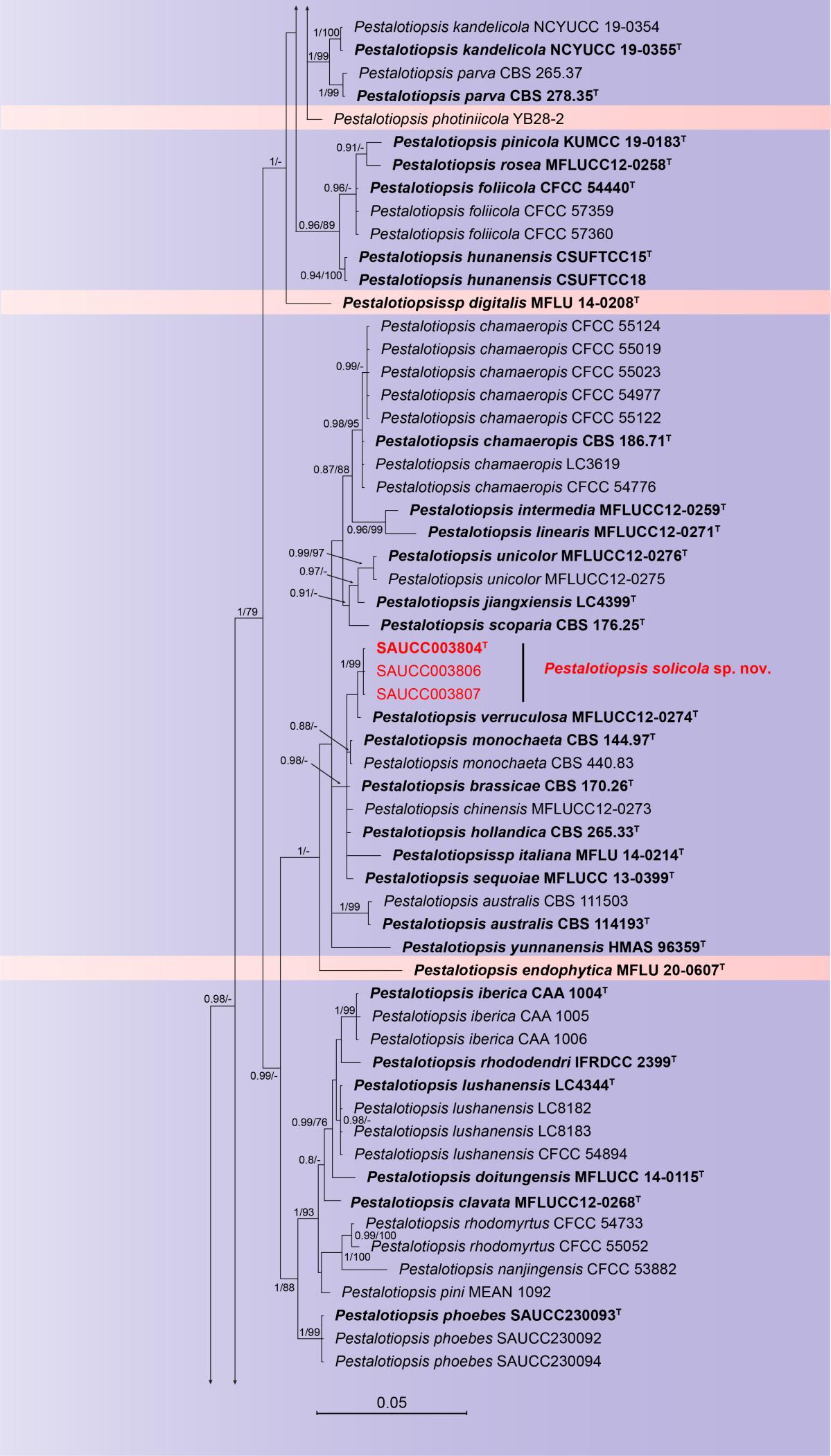
**

**Figure S2 Continued.**


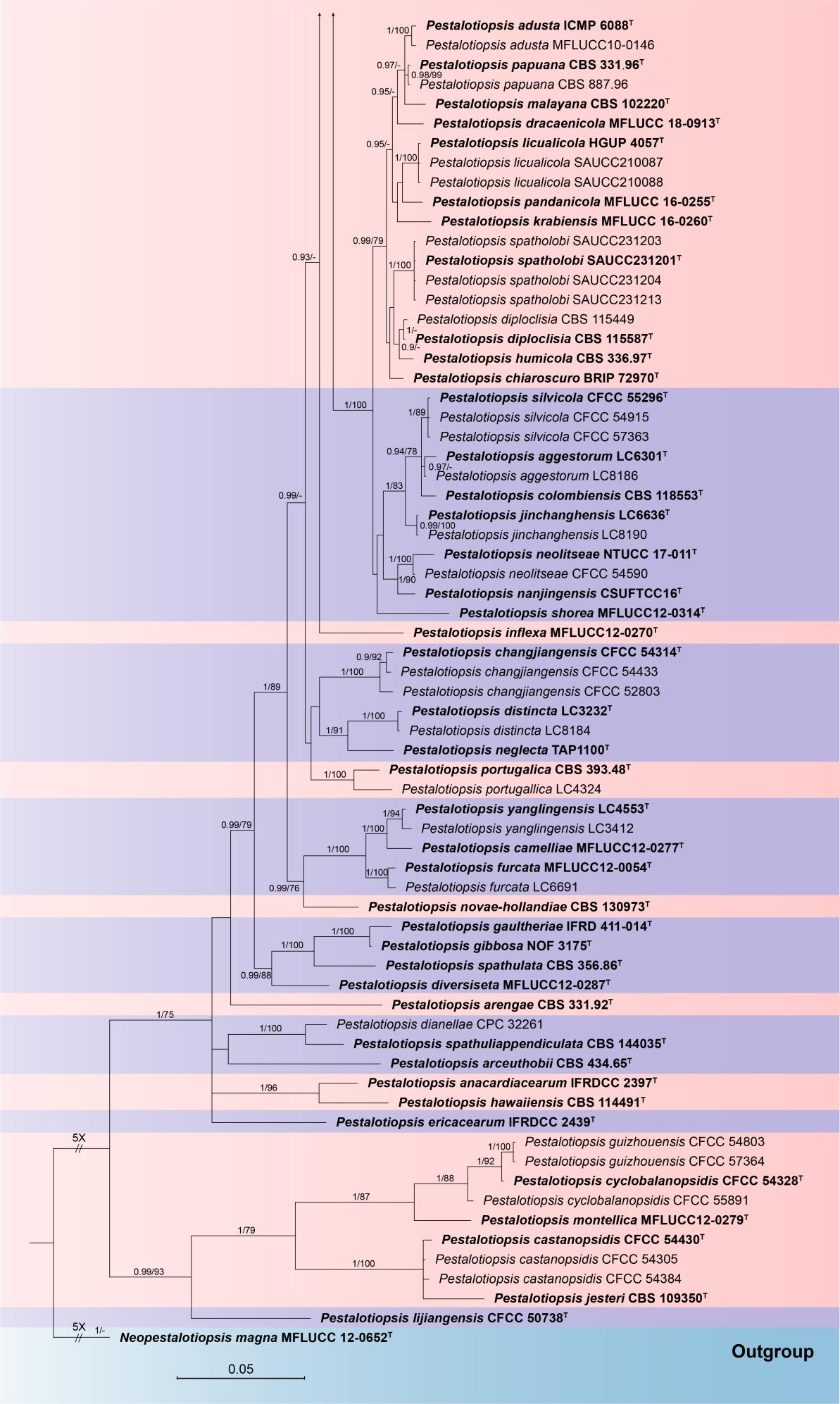


**Figure S2 Continued.**
