# Supplementary figures and images for: ﻿Three new microfungi (Ascomycota) species from southern China
Source: MycoKeys. 2024 Dec 11;111:87–110. doi: 10.3897/mycokeys.111.136483 (PMC11656163; doi:10.3897/mycokeys.111.136483)

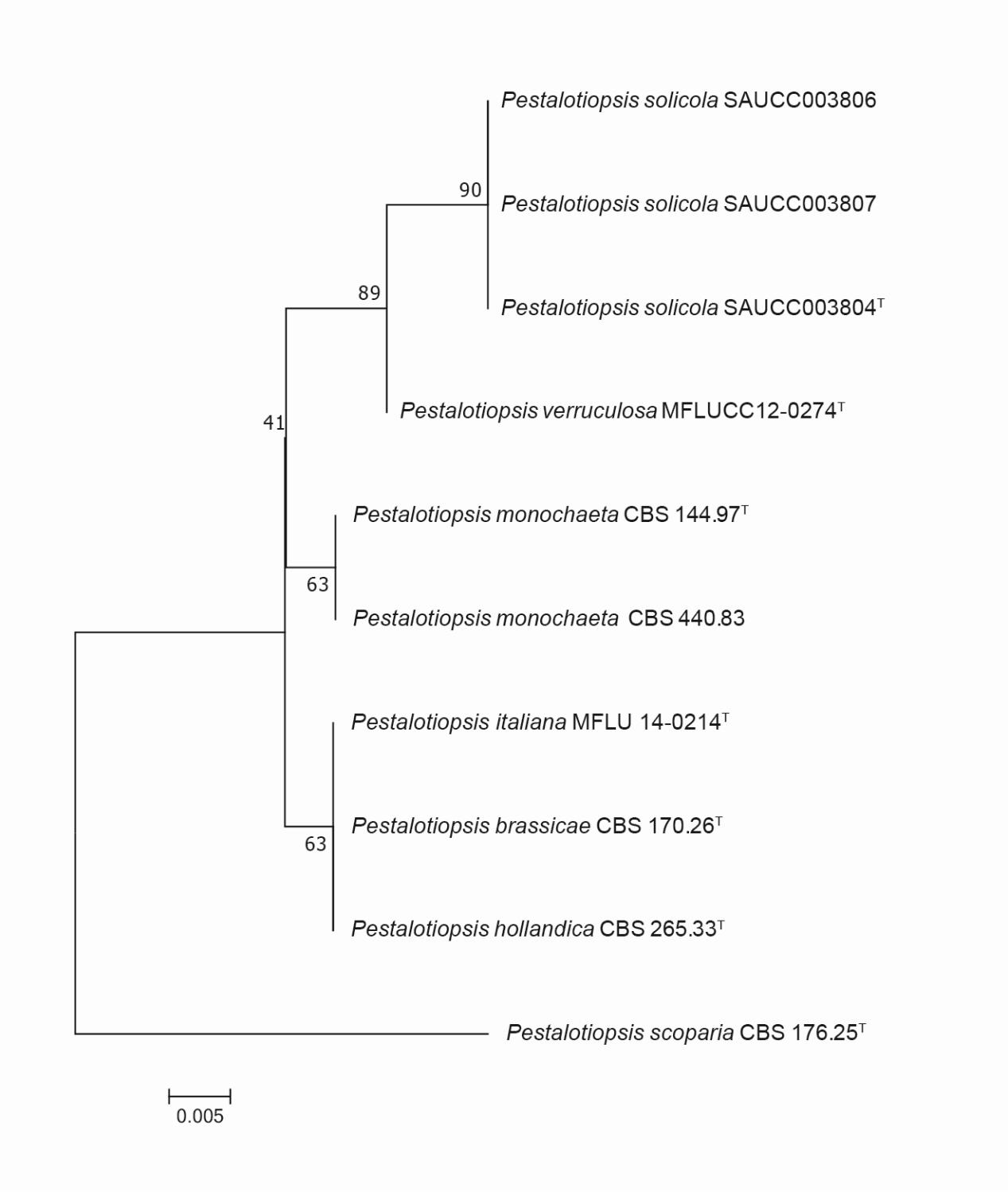


**Figure S3.** Phylogenetic tree of *Pestalotiopsis solicola* and related species based on TEF1α.

Supplement: Supplementary material 3 — Phylogenetic tree of Pestalotiopsissolicola and related species based on TEF1α [file mycokeys-111-087-s003.docx]

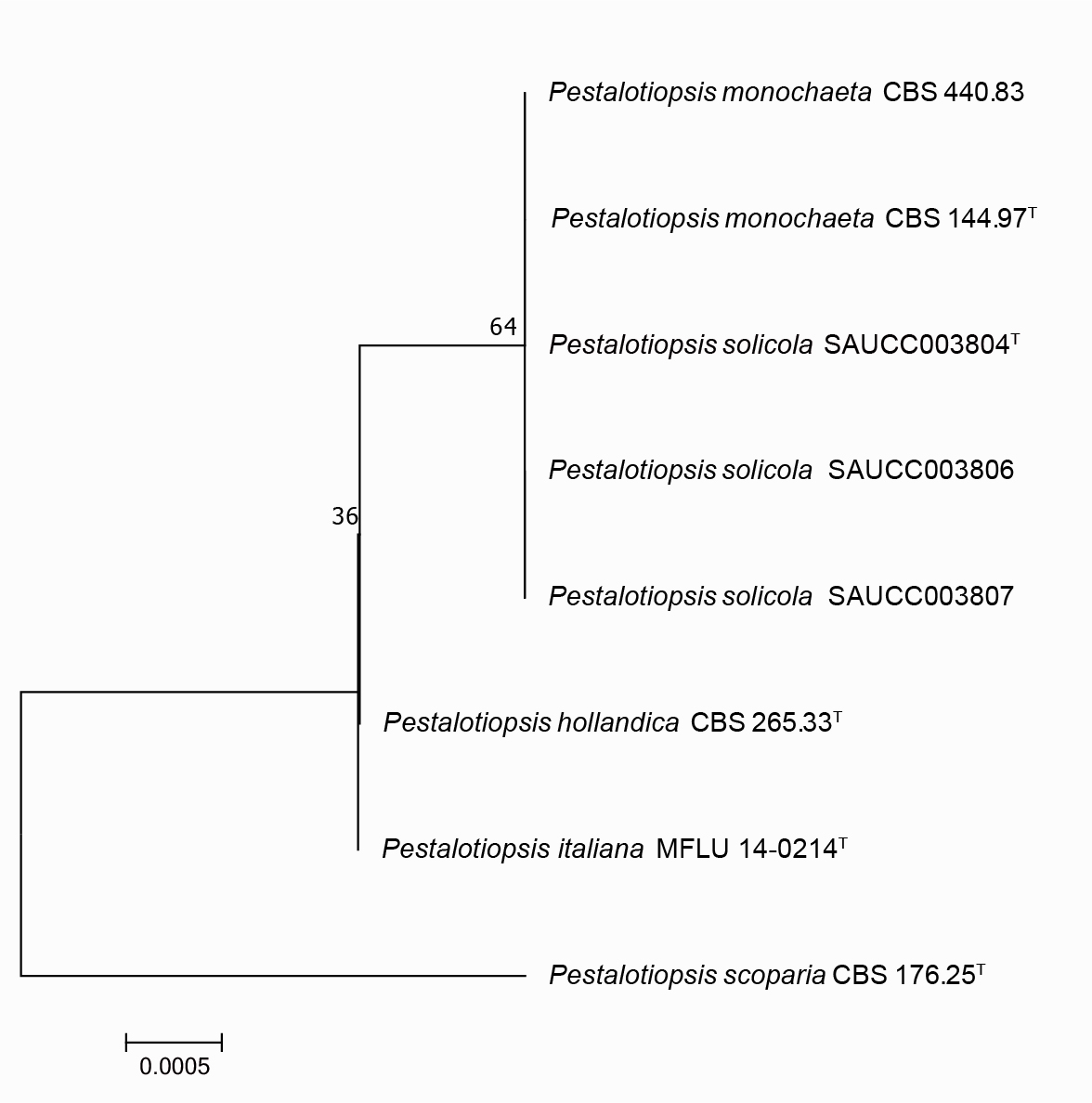


**Figure S4.** Phylogenetic tree of *Pestalotiopsis solicola* and related species based on TUB2.

Supplement: Supplementary material 4 — Phylogenetic tree of Pestalotiopsissolicola and related species based on TUB2 [file mycokeys-111-087-s004.docx]
