# Supplementary material for: ﻿Three new microfungi (Ascomycota) species from southern China
Source: MycoKeys. 2024 Dec 11;111:87–110. doi: 10.3897/mycokeys.111.136483 (PMC11656163; doi:10.3897/mycokeys.111.136483)
Supplement: Supplementary material 5 — GenBank accession numbers of the taxa used in Apiospora phylogenetic reconstruction [file mycokeys-111-087-s005.docx]

Table S1. GenBank accession numbers of the taxa used in *Apiospora* phylogenetic reconstruction.

| Species | Strain No. | Region | GenBank Accession No. | | | |
| --- | --- | --- | --- | --- | --- | --- |
|  |  |  | ITS | LSU | TEF1α | TUB2 |
| Arthrinium caricicola | CBS 145127 | China | MK014871 | MK014838 | MK017948 | MK017977 |
| *Apiospora acutiapica* | KUMCC 20-0210^T^ | China | MT946343 | MT946339 | MT947360 | MT947366 |
| *A. adinandrae* | SAUCC 1282B-1^T^ | China | OR739431 | OR739572 | OR753448 | OR757128 |
|  | SAUCC 1282B-2 | China | OR739432 | OR739573 | OR753449 | OR757129 |
| A. agari | KUC21333^T^ | Korea | MH498520 | MH498440 | MH544663 | MH498478 |
| A. aquatica | S-642^T^ | China | MK828608 | MK835806 | – | – |
| A. arctoscopi | KUC21331^T^ | Korea | MH498529 | MH498449 | MN868918 | MH498487 |
| A. arundinis | CBS 124788 | Switzerland | KF144885 | KF144929 | KF145017 | KF144975 |
| *A. aurea* | CBS 244.83^T^ | Spain | AB220251 | KF144935 | KF145023 | KF144981 |
| A. balearica | CBS 145129^T^ | Spain | MK014869 | MK014836 | MK017946 | MK017975 |
| **A. bambusigena** | **SAUCC 2446-2**T | **China** | **PP702396** | **PP711785** | **PP716797** | **PP716801** |
|  | **SAUCC 2446-6** | **China** | **PP702397** | **PP711786** | **PP716798** | **PP716802** |
| A. bambusicola | MFLUCC 20-0144^T^ | Thailand | MW173030 | MW173087 | MW183262 | – |
| *A. bawanglingensis* | SAUCC BW0444^T^ | China | OR739429 | OR739570 | OR753446 | OR757126 |
|  | SAUCC BW04441 | China | OQ592551 | OQ615280 | OQ613324 | OQ613302 |
| A. biserialis | CGMCC3.20135^T^ | China | MW481708 | MW478885 | MW522938 | MW522955 |
| A. camelliae-sinensis | LC5007^T^ | China | KY494704 | KY494780 | KY705103 | KY705173 |
| A. chiangraiense | MFLUCC 21-0053^T^ | Thailand | MZ542520 | MZ542524 | – | MZ546409 |
| A. chromolaenae | MFLUCC 17-1505^T^ | Thailand | MT214342 | MT214436 | – | – |
| *A. cordylines* | GUCC 10027^T^ | China | MT040106 | – | MT040127 | MT040148 |
| A. cyclobalanopsidis | CGMCC3.20136^T^ | China | MW481713 | MW478892 | MW522945 | MW522962 |
| A. descalsii | CBS 145130^T^ | Spain | MK014870 | MK014837 | MK017947 | MK017976 |
| A. dichotomanthi | LC4950^T^ | China | KY494697 | KY494773 | KY705096 | KY705167 |
| *A. dongyingensis* | SAUCC 0302^T^ | China | OP563375 | OP572424 | OP573264 | OP573270 |
|  | SAUCC 0303 | China | OP563374 | OP572423 | OP573263 | OP573269 |
| A. esporlensis | CBS 145136^T^ | Spain | MK014878 | MK014845 | MK017954 | MK017983 |
| A. euphorbiae | IMI 285638b | Bangladesh | AB220241 | AB220335 | – | AB220288 |
| A. fermenti | KUC21289^T^ | Korea | MF615226 | MF615213 | MH544667 | MF615231 |
| A. gaoyouensis | CFCC 52301^T^ | China | MH197124 | – | MH236793 | MH236789 |
| A. garethjonesii | JHB004^T^ | China | KY356086 | KY356091 | – | – |
| *A. gelatinosa* | HKAS 111962^T^ | China | MW481706 | MW478888 | MW522941 | MW522958 |
| A. guiyangensis | HKAS 102403^T^ | China | MW240647 | MW240577 | MW759535 | MW775604 |
| A. guizhouensis | LC5322^T^ | China | KY494709 | KY494785 | KY705108 | KY705178 |
| *A. hainanensis* | SAUCC 1681^T^ | China | OP563373 | OP572422 | OP573262 | OP573268 |
|  | SAUCC 1682 | China | OP563372 | OP572421 | OP573261 | OP573267 |
| *A. hispanica* | IMI 326877^T^ | Spain | AB220242 | AB220336 | – | AB220289 |
| A. hydei | CBS 114990^T^ | China | KF144890 | KF144936 | KF145024 | KF144982 |
| A. hyphopodii | MFLUCC 15-003^T^ | Thailand | KR069110 | – | – | – |
| A. hysterina | ICPM 6889^T^ | New Zealand | MK014874 | MK014841 | MK017951 | MK017980 |
| A. iberica | AP10118^T^ | Portugal | MK014879 | MK014846 | MK017955 | MK017984 |
| *A. indocalami* | SAUCC BW0455^T^ | China | OR739430 | OR739571 | OR753447 | OR757127 |
|  | SAUCC BW04551 | China | OQ592550 | OQ615279 | OQ613323 | OQ613301 |
| A. intestini | CBS 135835^T^ | India | KR011352 | KR149063 | KR011351 | KR011350 |
| A. italica | CBS 145138^T^ | Italy | MK014880 | MK014847 | MK017956 | MK017985 |
| A. jatrophae | CBS 134262^T^ | India | JQ246355 | – | – | – |
| A. jiangxiensis | LC4577^T^ | China | KY494693 | KY494769 | KY705092 | KY705163 |
| A. kogelbergensis | CBS 113333^T^ | South Africa | KF144892 | KF144938 | KF145026 | KF144984 |
| *A. koreana* | KUC21332^T^ | Korea | MH498524 | MH498444 | MH544664 | MH498482 |
| A. locuta-pollinis | LC11683^T^ | China | MF939595 | – | MF939616 | MF939622 |
| A. longistroma | MFLUCC 11-0481^T^ | Thailand | KU940141 | KU863129 | – | – |
| A. malaysiana | CBS 102053^T^ | Malaysia | KF144896 | KF144942 | KF145030 | KF144988 |
| *A. machili* | SAUCC 1175A-4 | China | OR739433 | OR739574 | OR753450 | OR757130 |
|  | SAUCC 1175 | China | OQ592560 | OQ615289 | OQ613333 | OQ613307 |
| A. marianiae | AP18219^T^ | Spain | ON692406 | ON692422 | ON677180 | ON677186 |
| A. marii | CBS 497.90^T^ | Spain | MH873913 | KF144947 | KF145035 | KF144993 |
| *A. marina* | KUC21328^T^ | Korea | MH498538 | MH498458 | MH544669 | MH498496 |
| *A. mediterranea* | IMI 326875^T^ | Spain | AB220243 | AB220337 | – | AB220290 |
| *A. minutispora* | 17E-042^T^ | South Korea | LC517882 | – | LC518889 | LC518888 |
| *A. montagnei* | AP301120^T^ | Spain | ON692408 | ON692424 | ON677182 | ON677188 |
|  | AP19421 | Spain | ON692418 | ON692425 | ON677183 | ON677189 |
|  | CPC 18900 | Italy | KF144909 | KF144956 | KF145043 | KF145001 |
| A. mori | MFLU 18-2514^T^ | China | MW114313 | MW114393 | – | – |
| A. multiloculata | MFLUCC 21-0023^T^ | Thailand | OL873137 | OL873138 | – | OL874718 |
| A. mytilomorpha | DAOM 214595^T^ | India | KY494685 | – | – | – |
| A. neobambusae | LC7106^T^ | China | KY494718 | KY494794 | KY806204 | KY705186 |
| A. neochinense | CFCC 53036^T^ | China | MK819291 | – | MK818545 | MK818547 |
| A. neogarethjonesii | HKAS 102408^T^ | China | MK070897 | MK070898 | – | – |
| A. neosubglobosa | KUMCC 16-0203^T^ | China | KY356090 | KY356095 | – | – |
| A. obovata | LC4940^T^ | China | KY494696 | KY494772 | KY705095 | KY705166 |
| A. ovata | CBS 115042^T^ | China | KF144903 | KF144950 | KF145037 | KF144995 |
| A. paraphaeosperma | MFLUCC 13-0644^T^ | Thailand | KX822128 | KX822124 | – | – |
| *A. phyllostachydis* | MFLUCC 18-1101^T^ | China | MK351842 | MH368077 | MK340918 | MK291949 |
| A. piptatheri | CBS 145149^T^ | Spain | MK014893 | MK014860 | MK017969 | – |
| *A. pseudomarii* | GUCC 10228^T^ | China | MT040124 | – | MT040145 | MT040166 |
| A. pseudoparenchymatica | LC7234^T^ | China | KY494743 | KY494819 | KY705139 | KY705211 |
| A. pseudorasikravindrae | KUMCC 20-0208^T^ | China | MT946344 | – | MT947361 | MT947367 |
| A. pseudosinensis | CPC 21546^T^ | Netherlands | KF144910 | KF144957 | KF145044 | MN868936 |
| A. pseudospegazzinii | CBS 102052^T^ | Malaysia | KF144911 | KF144958 | KF145045 | KF145002 |
| A. pterosperma | CPC 20193^T^ | Australia | KF144913 | KF144960 | KF145046 | KF145004 |
| A. pusillisperma | KUC21321^T^ | Korea | MH498533 | MH498453 | MN868930 | MH498491 |
| A. qinlingensis | CFCC 52303^T^ | China | MH197120 | – | MH236795 | MH236791 |
| A. rasikravindrae | LC5449 | China | KY494713 | KY494789 | KY705112 | KY705182 |
| A. sacchari | CBS 212.30 | UK | KF144916 | KF144962 | KF145047 | KF145005 |
| *A. saccharicola* | CBS 191.73 | Netherlands | KF144920 | KF144966 | KF145051 | KF145009 |
| A. sargassi | KUC21228^T^ | Korea | KT207746 | KT207696 | MH544677 | KT207644 |
| A. sasae | CBS 146808^T^ | Netherlands | MW883402 | MW883797 | MW890104 | MW890120 |
| A. septata | CGMCC 3.20134^T^ | China | MW481711 | MW478890 | MW522943 | MW522960 |
| *A. serenensis* | IMI 326869^T^ | Spain | AB220250 | AB220344 | – | AB220297 |
| A. setariae | CFCC 54041^T^ | China | MT492004 | – | – | – |
| *A. setostroma* | KUMCC 19-0217 | China | MN528012 | MN528011 | MN527357 | – |
| A. sichuanensis | HKAS 107008^T^ | China | MW240648 | MW240578 | MW759536 | MW775605 |
| A. sorghi | URM 93000^T^ | Brazil | MK371706 | – | – | MK348526 |
| *A. sphaerosperma* | CBS 114314 | Iran | KF144904 | KF144951 | KF145038 | KF144996 |
| *A. stipae* | CBS 146804^T^ | Spain | MW883403 | MW883798 | MW890082 | MW890121 |
| *A. subglobosa* | MFLUCC 11-0397 | Thailand | KR069112 | KR069113 | – | – |
| *A. subrosea* | LC7292^T^ | China | KY494752 | KY494828 | KY705148 | KY705220 |
| A. taeanense | KUC21322^T^ | South Korea | MH498515 | – | MH544662 | MH498473 |
| A. thailandica | LC5630 | China | KY494714 | KY494790 | KY705113 | KY806200 |
| A. vietnamensis | IMI 99670^T^ | Vietnam | KX986096 | KX986111 | – | KY019466 |
| A. xenocordella | CBS 478.86^T^ | Zimbabwe | KF144925 | KF144970 | KF145055 | KF145013 |
| A. yunnana | MFLUCC 15-0002^T^ | China | KU940147 | KU863135 | – | – |

Notes: Ex-type or ex-epitype strains are marked with “T” and the new species information described in this study is marked in bold.
