# Supplementary material for: ﻿Three new microfungi (Ascomycota) species from southern China
Source: MycoKeys. 2024 Dec 11;111:87–110. doi: 10.3897/mycokeys.111.136483 (PMC11656163; doi:10.3897/mycokeys.111.136483)
Supplement: Supplementary material 6 — GenBank accession numbers of the taxa used in Microdochium phylogenetic reconstruction [file mycokeys-111-087-s006.docx]

Table S2. GenBank accession numbers of the taxa used in *Microdochium* phylogenetic reconstruction.

| Species | Strain No. | Region | GenBank Accession No. | | | |
| --- | --- | --- | --- | --- | --- | --- |
|  |  |  | ITS | LSU | RPB2 | TUB2 |
| *Idriela lunata* | CBS 204.56T | USA | KP859044 | KP858981 | – | – |
|  | CBS 177.57 | USA | KP859043 | KP858980 | – | – |
| *Microdochium albescens* | CBS 243.83 | Ivory Coast | KP858994 | KP858930 | KP859103 | KP859057 |
|  | CBS 291.79 | Ivory Coast | KP858996 | KP858932 | KP859105 | KP859059 |
| *M. bambusae* | SAUCC 1862-1T | China | OR702567 | OR702576 | OR715785 | PP445175 |
|  | SAUCC 1866-1 | China | OR702568 | OR702577 | OR715786 | PP445176 |
| *M. bolleyi* | CBS 540.92 | Syria | KP859010 | KP858946 | KP859119 | KP859073 |
| *M. chrysanthemoides* | CGMCC 3.17929T | China | KU746690 | KU746736 | – | – |
| *M. chuxiongense* | YFCC 8794^T^ | China | OK586161 | OK586160 | OK584019 | OK556901 |
| *M. citrinidiscum* | CBS 109067T | Peru | KP859003 | KP858939 | KP859112 | KP859066 |
| *M. colombiense* | CBS 624.94 | Colombia | KP858999 | KP858935 | KP859108 | KP859062 |
| *M. dawsoniorum* | BRIP 65649T | Australia | MK966337 | – | – | – |
| *M. ﬁsheri* | CBS 242.90T | UK | KP859015 | KP858951 | KP859124 | KP859078 |
| *M. graminearum* | CGMCC 3.23525T | China | OP103966 | OP104016 | OP236027 | – |
|  | CGMCC 3.23524 | China | OP103965 | OP104015 | OP236026 | – |
| *M. hainanense* | SAUCC 210782 | China | OM956296 | OM959324 | OM981154 | OM981147 |
|  | SAUCC 210781T | China | OM956295 | OM959323 | OM981153 | OM981146 |
| *M. indocalami* | SAUCC 1016^T^ | China | MT199884 | MT199878 | MT510550 | MT435653 |
| ***M. jianfenglingense*** | **SAUCC 1862-2^T^** | **China** | **PP702394** | **PP711783** | **PP716793** | **PP716799** |
|  | **SAUCC 1862-5** | **China** | **PP702395** | **PP711784** | **PP716794** | **PP716800** |
| *M. lycopodinum* | CBS 146.68 | The Netherlands | KP858993 | KP858929 | KP859102 | KP859056 |
|  | CBS 122885T | Germany | KP859016 | KP858952 | KP859125 | KP859080 |
| *M. maculosum* | COAD 3358T | Brazil | Ok966954 | Ok966953 | – | – |
| *M. majus* | CBS 741.79 | Germany | KP859001 | KP858937 | KP859110 | KP859064 |
| *M. miscanthi* | SAUCC 211092T | China | OM956214 | OM957532 | OM981148 | OM981141 |
|  | SAUCC 211093 | China | OM956215 | OM957533 | OM981149 | OM981142 |
| *M. musae* | CBS 143499 | Malaysia | MH107894 | MH107941 | – | – |
|  | CBS 143500T | Malaysia | MH107895 | MH107942 | MH108003 | – |
| *M. nannuoshanense* | SAUCC 2450-1T | China | OR702569 | OR702578 | OR715787 | PP445177 |
|  | SAUCC 2450-3 | China | OR702570 | OR702579 | OR715788 | PP445178 |
| *M. neoqueenslandicum* | CBS 445.95 | The Netherlands | KP858997 | KP858933 | KP859106 | KP859060 |
|  | CBS 108926T | The Netherlands | KP859002 | KP858938 | KP859111 | KP859065 |
| *M. nivale* | CBS 116205T | UK | KP859008 | KP858944 | KP859117 | KP859071 |
| *M. nivale var. majus* | CBS 177.29 | The Netherlands | MH855031 | MH866500 | – | – |
| *M. nivale var. nivales* | CBS 288.50 | The Netherlands | – | MH868135 | – | – |
| *M. novae-zelandiae* | CPC 29376T | The Netherlands | LT990655 | – | LT990641 | LT990608 |
|  | CPC 29693 | The Netherlands | LT990656 | – | LT990642 | LT990609 |
| *M. paspali* | HK-ML-1371 | China | KJ569509 | – | – | KJ569514 |
|  | CBS 138620T | China | KJ569513 | – | – | KJ569518 |
| *M. phyllosaprophyticum* | SAUCC 3583-1T | China | OR702571 | OR702580 | OR715789 | PP445179 |
|  | SAUCC 3583-6 | China | OR702572 | OR702581 | OR715790 | PP445180 |
| *M. phragmitis* | CBS 285.71T | Poland | KP859013 | KP858949 | KP859122 | KP859077 |
|  | CBS 423.78 | Poland | KP859012 | KP858948 | KP859121 | KP859076 |
| *M. poae* | CGMCC 3.19170T | China | MH740898 |  | MH740906 | MH740914 |
|  | LC 12115 | China | MH740901 |  | MH740909 | MH740917 |
|  | LC 12116 | China | MH740902 | – | MH740910 | MH740918 |
| *M. ratticaudae* | BRIP 68298T | Australia | MW481661 | MW481666 | MW626890 | – |
| *M. rhopalostylidis* | CBS 145125T | The Netherlands | MK442592 | MK442532 | MK442667 |  |
| *M. seminicola* | CBS 139951T | Switzerland | KP859038 | KP858974 | KP859147 | KP859101 |
|  | CPC 26001 | Canada | KP859025 | KP858961 | KP859134 | KP859088 |
|  | DAOM 250161 | Canada | KP859034 | KP858970 | KP859143 | KP859097 |
| *M. shilinense* | CGMCC 3.23531T | China | OP103972 | OP104022 | – | OP242834 |
| *M. sinense* | SAUCC 211097T | China | OM956289 | OM959225 | OM981151 | OM981144 |
|  | SAUCC 211098 | China | OM956290 | OM959226 | OM981152 | OM981145 |
| *M. sorghi* | CBS 691.96 | Cuba | KP859000 | KP858936 | KP859109 | KP859063 |
| *M. tainanense* | CBS 269.76T | The Netherlands | KP859009 | KP858945 | KP859118 | KP859072 |
|  | CBS 270.76 | The Netherlands | KP858995 | KP858931 | KP859104 | KP859058 |
| *M. trichocladiopsis* | CBS 623.77T | The Netherlands | KP858998 | KP858934 | KP859107 | KP859061 |
| *M. yunnanense* | SAUCC 1011T | China | MT199881 | MT199875 | MT510547 | MT435650 |
|  | SAUCC 1012 | China | MT199882 | MT199876 | MT510548 | MT435651 |

Notes: Ex-type or ex-epitype strains are marked with “T” and the new species information described in this study is marked in bold.
