# Supplementary material for: ﻿Three new microfungi (Ascomycota) species from southern China
Source: MycoKeys. 2024 Dec 11;111:87–110. doi: 10.3897/mycokeys.111.136483 (PMC11656163; doi:10.3897/mycokeys.111.136483)
Supplement: Supplementary material 7 — GenBank accession numbers of the taxa used in Pestalotiopsis phylogenetic reconstruction [file mycokeys-111-087-s007.docx]

Table S3. GenBank accession numbers of the taxa used in *Pestalotiopsis* phylogenetic reconstruction.

| Species | Strain No. | Region | GenBank Accession No. | | |
| --- | --- | --- | --- | --- | --- |
|  |  |  | ITS | TEF1α | TUB2 |
| *Neopestalotiopsis magna* | MFLUCC 12-0652^T^ | France | KF582795 | KF582791 | KF582793 |
| *Pestalotiopsis abietis* | CFCC 53011^T^ | China | MK397013 | MK622277 | MK622280 |
|  | CFCC 53012 | China | MK397014 | MK622278 | MK622281 |
|  | CFCC 53013 | China | MK397015 | MK622279 | MK622282 |
| *P. adusta* | ICMP 6088^T^ | Fiji | JX399006 | JX399070 | JX399037 |
|  | MFLUCC10-146 | Thailand | JX399007 | JX399071 | JX399038 |
| *P. aggestorum* | LC6301^T^ | China | KX895015 | KX895234 | KX895348 |
|  | LC8186 | China | KY464140 | KY464150 | KY464160 |
| *P. anacardiacearum* | IFRDCC 2397^T^ | China | KC247154 | KC247156 | KC247155 |
| *P. anhuiensis* | CFCC 54791^T^ | China | ON007028 | ON005045 | ON005056 |
| *P. arceuthobii* | CBS 434.65^T^ | USA | KM199341 | KM199516 | KM199427 |
| *P. arengae* | CBS 331.92^T^ | Singapore | KM199340 | KM199515 | KM199426 |
| *P. australasiae* | CBS 114126^T^ | New Zealand | KM199297 | KM199499 | KM199409 |
|  | CBS 114141 | New South Wales | KM199298 | KM199501 | KM199410 |
| *P. australis* | CBS 111503 | South Africa | KM199331 | KM199557 | KM199382 |
|  | CBS 114193^T^ | New South Wales | KM199332 | KM199475 | KM199383 |
| *P. biciliata* | CBS 124463^T^ | Slovakia | KM199308 | KM199505 | KM199399 |
|  | CBS 236.38 | Italy | KM199309 | KM199506 | KM199401 |
| *P. brachiata* | LC2988^T^ | China | KX894933 | KX895150 | KX895265 |
|  | LC8188 | China | KY464142 | KY464152 | KY464162 |
|  | LC8189 | China | KY464143 | KY464153 | KY464163 |
| *P. brassicae* | CBS 170.26^T^ | New Zealand | KM199379 | KM199558 | – |
| *P. camelliae* | MFLUCC12-0277^T^ | China | JX399010 | JX399074 | JX399041 |
| *P. camelliae-oleiferae* | CSUFTCC08^T^ | China | OK493593 | OK507963 | OK562368 |
|  | CSUFTCC09 | China | OK493594 | OK507964 | OK562369 |
| *P. castanopsidis* | CFCC 54430^T^ | China | OK339732 | OK358493 | OK358508 |
|  | CFCC 54305 | China | OK339733 | OK358494 | OK358509 |
|  | CFCC 54384 | China | OK339734 | OK358495 | OK358510 |
| *P. chamaeropis* | CBS 186.71^T^ | Italy | KM199326 | KM199473 | KM199391 |
|  | LC3619 | China | KX894991 | KX895208 | KX895322 |
|  | CFCC 55124 | China | OM746221 | OM839993 | OM839894 |
|  | CFCC 55019 | China | OM746224 | OM839996 | OM839897 |
|  | CFCC 55023 | China | OM746233 | OM840005 | OM839906 |
|  | CFCC 54977 | China | OM746223 | OM839995 | OM839896 |
|  | CFCC 55122 | China | OM746229 | OM840001 | OM839902 |
|  | CFCC 54776 | China | OM746234 | OM840006 | OM839907 |
| *P. changjiangensis* | CFCC 54314^T^ | China | OK339739 | OK358500 | OK358515 |
|  | CFCC 54433 | China | OK339740 | OK358501 | OK358516 |
|  | CFCC 52803 | China | OK339741 | OK358502 | OK358517 |
| *P. chiaroscuro* | BRIP 72970^T^ | Australia | OK422510 | OK423753 | OK423752 |
| *P. chinensis* | MFLUCC12-0273 | China | JX398995 | – | – |
| *P. clavata* | MFLUCC12-0268^T^ | China | JX398990 | JX399056 | JX399025 |
| *P. colombiensis* | CBS 118553^T^ | Colombia | KM199307 | KM199488 | KM199421 |
| *P. cyclobalanopsidis* | CFCC 54328^T^ | China | OK339735 | OK358496 | OK358511 |
|  | CFCC 55891 | China | OK339736 | OK358497 | OK358512 |
| *P. dianellae* | CPC 32261 | Australia | MG386051 | – | MG386164 |
| *P. digitalis* | MFLU 14-0208^T^ | New Zealand | KP781879 | – | KP781883 |
| *P. diploclisiae* | CBS 115449 | China | KM199314 | KM199485 | KM199416 |
|  | CBS 115587^T^ | China | KM199320 | KM199486 | KM199419 |
| *P. disseminata* | CBS 143904 | New Zealand | MH554152 | MH554587 | MH554825 |
|  | MEAN 1165 | Portugal | MT374687 | MT374699 | MT374712 |
| *P. distincta* | LC3232^T^ | China | KX894961 | KX895178 | KX895293 |
|  | LC8184 | China | KY464138 | KY464148 | KY464158 |
| *P. diversiseta* | MFLUCC12-0287^T^ | China | JX399009 | JX399073 | JX399040 |
| *P. doitungensis* | MFLUCC 14-0115^T^ | Thailand | MK993574 | MK975832 | MK975837 |
| *P. dracaenicola* | MFLUCC 18-0913^T^ | Thailand | MN962731 | MN962732 | MN962733 |
| *P. dracontomelonis* | MFLU 14-0207^T^ | Thailand | KP781877 | KP781880 | – |
| *P. endophytica* | MFLU 20-0607^T^ | Thailand | MW263946 | MW417119 | – |
| *P. ericacearum* | IFRDCC 2439^T^ | China | KC537807 | KC537814 | KC537821 |
| *P. etonensis* | BRIP 66615^T^ | Australia | MK966339 | MK977635 | MK977634 |
| *P. ficicola* | SAUCC230046^T^ | China | OQ691974 | OQ718691 | OQ718749 |
|  | SAUCC230042 | China | OQ691972 | OQ718689 | OQ718747 |
|  | SAUCC230043 | China | OQ691973 | OQ718690 | OQ718748 |
| *P. foliicola* | CFCC 54440^T^ | China | ON007029 | ON005046 | ON005057 |
|  | CFCC 57359 | China | ON007030 | ON005047 | ON005058 |
|  | CFCC 57360 | China | ON007031 | ON005048 | ON005059 |
| *P. formosana* | NTUCC 17-009 | China | MH809381 | MH809389 | MH809385 |
| *P. furcata* | MFLUCC12-0054^T^ | Thailand | JQ683724 | JQ683740 | JQ683708 |
|  | LC6691 | China | KX895030 | KX895248 | KX895363 |
| *P. gaultheriae* | IFRD 411-014^T^ | China | KC537805 | KC537812 | KC537819 |
| *P. gibbosa* | NOF 3175^T^ | Canada | LC311589 | LC311591 | LC311590 |
| *P. grandis-urophylla* | E-72-02 | Brazil | KU926708 | KU926712 | KU926716 |
|  | E-72-03 | Brazil | KU926709 | KU926713 | KU926717 |
|  | E-72-04 | Brazil | KU926710 | KU926714 | KU926718 |
|  | E-72-06 | Brazil | KU926711 | KU926715 | KU926719 |
| *P. grevilleae* | CBS 114127^T^ | Australia | KM199300 | KM199504 | KM199407 |
| *P. guangxiensis* | CFCC 54308^T^ | China | OK339737 | OK358498 | OK358513 |
|  | CFCC 54300 | China | OK339738 | OK358499 | OK358514 |
| *P. guizhouensis* | CFCC 54803 | China | ON007035 | ON005052 | ON005063 |
|  | CFCC 57364 | China | ON007036 | ON005053 | ON005064 |
| *P. hawaiiensis* | CBS 114491^T^ | USA | KM199339 | KM199514 | KM199428 |
| *P. hispanica* | CBS 115391^T^ | Spain | MH553981 | MH554399 | MH554640 |
| *P. hollandica* | CBS 265.33^T^ | Netherlands | KM199328 | KM199481 | KM199388 |
| *P. humicola* | CBS 336.97^T^ | Papua New Guinea | KM199317 | KM199484 | KM199420 |
| *P. hunanensis* | CSUFTCC15^T^ | China | OK493599 | OK507969 | OK562374 |
|  | CSUFTCC18 | China | OK493600 | OK507970 | OK562375 |
| *P. hydei* | MFLUCC 20-0135 | Thailand | MW266063 | MW251113 | MW251112 |
| *P. iberica* | CAA 1004^T^ | Spain | MW732248 | MW759038 | MW759035 |
|  | CAA 1005 | Spain | MW732250 | MW759037 | MW759034 |
|  | CAA 1006 | Spain | MW732249 | MW759039 | MW759036 |
| *P. inflexa* | MFLUCC12-0270^T^ | China | JX399008 | JX399072 | JX399039 |
| *P. intermedia* | MFLUCC12-0259^T^ | China | JX398993 | JX399059 | JX399028 |
| *P. italiana* | MFLU 14-0214^T^ | Italy | KP781878 | KP781881 | KP781882 |
| *P. jesteri* | CBS 109350^T^ | Papua New Guinea | KM199380 | KM199554 | – |
| *P. jiangxiensis* | LC4399^T^ | China | KX895009 | KX895227 | KX895341 |
| *P. jinchanghensis* | LC6636^T^ | China | KX895028 | KX895247 | KX895361 |
|  | LC8190 | China | KY464144 | KY464154 | KY464164 |
| *P. kaki* | KNU-PT-1804^T^ | Korea | LC552953 | LC553555 | LC552954 |
| *P. kandelicola* | NCYUCC 19-0354 | China | MT560723 | MT563102 | MT563100 |
|  | NCYUCC 19-0355^T^ | China | MT560722 | MT563101 | MT563099 |
| *P. kenyana* | CBS 442.67^T^ | Kenya | KM199302 | KM199502 | KM199395 |
|  | LC6633 | China | KX895027 | KX895246 | KX895360 |
| *P. knightiae* | CBS 111963 | New Zealand | KM199311 | KM199495 | KM199406 |
|  | CBS 114138^T^ | New Zealand | KM199310 | KM199497 | KM199408 |
| *P. krabiensis* | MFLUCC 16-0260^T^ | Thailand | MH388360 | MH388395 | MH412722 |
| *P. leucadendri* | CBS 121417^T^ | South Africa | MH553987 | MH554412 | MH554654 |
| *P. licualicola* | HGUP 4057^T^ | China | KC492509 | KC481684 | KC481683 |
|  | SAUCC210087 | China | OK087323 | OK104879 | OK104872 |
|  | SAUCC210088 | China | OK087324 | OK104880 | OK104873 |
| *P. lijiangensis* | CFCC 50738^T^ | China | KU860520 | KU844185 | – |
| *P. linearis* | MFLUCC12-0271^T^ | China | JX398992 | JX399058 | JX399027 |
| *P. lithocarpi* | CFCC 55100^T^ | China | OK339742 | OK358503 | OK358518 |
|  | CFCC 55893 | China | OK339743 | OK358504 | OK358519 |
| *P. lushanensis* | LC4344^T^ | China | KX895005 | KX895223 | KX895337 |
|  | LC8182 | China | KY464136 | KY464146 | KY464156 |
|  | LC8183 | China | KY464137 | KY464147 | KY464157 |
|  | CFCC 54894 | China | OM746282 | OM840054 | OM839955 |
| *P. macadamiae* | BRIP 63738b | Australia | KX186588 | KX186621 | KX186680 |
|  | BRIP 63739b | Australia | KX186587 | KX186620 | KX186679 |
|  | BRIP 63741a | Australia | KX186586 | KX186619 | KX186678 |
| *P. malayana* | CBS 102220^T^ | Malaysia | KM199306 | KM199482 | KM199411 |
| *P. microspora* | SS1-033I | Canada | MT644300 | – | – |
| *P. monochaeta* | CBS 144.97^T^ | Netherlands | KM199327 | KM199479 | KM199386 |
|  | CBS 440.83 | Netherlands | KM199329 | KM199480 | KM199387 |
| *P. montellica* | MFLUCC12-0279^T^ | China | JX399012 | JX399076 | JX399043 |
| *P. nanjingensis* | CFCC 53882 | China | OM746295 | OM840067 | OM839968 |
|  | CSUFTCC16^T^ | China | OK493602 | OK507972 | OK562377 |
| *P. nanningensis* | CSUFTCC10^T^ | China | OK493596 | OK507966 | OK562371 |
| *P. neglecta* | TAP1100^T^ | Japan | AB482220 | LC311600 | LC311599 |
| *P. neolitseae* | NTUCC 17-011^T^ | China | MH809383 | MH809391 | MH809387 |
|  | CFCC 54590 | China | OK339744 | OK358505 | OK358520 |
| *P. novae-hollandiae* | CBS 130973^T^ | Australia | KM199337 | KM199511 | KM199425 |
| *P. oryzae* | CBS 111522 | USA | KM199294 | KM199493 | KM199394 |
|  | CBS 171.26 | Italy | KM199304 | KM199494 | KM199397 |
|  | CBS 353.69^T^ | Denmark | KM199299 | KM199496 | KM199398 |
| *P. pandanicola* | MFLUCC 16-0255^T^ | Thailand | MH388361 | MH388396 | MH412723 |
| *P. papuana* | CBS 331.96^T^ | Papua New Guinea | KM199321 | KM199491 | KM199413 |
|  | CBS 887.96 | Papua New Guinea | KM199318 | KM199492 | KM199415 |
| *P. parva* | CBS 265.37 | NA | KM199312 | KM199508 | KM199404 |
|  | CBS 278.35^T^ | NA | KM199313 | KM199509 | KM199405 |
| *P. phoebes* | SAUCC230093^T^ | China | OQ692028 | OQ718745 | OQ718803 |
|  | SAUCC230092 | China | OQ692027 | OQ718744 | OQ718802 |
|  | SAUCC230094 | China | OQ692029 | OQ718746 | OQ718804 |
| *P. photiniicola* | YB28-2 | China | MK228997 | MK512491 | MK360938 |
| *P. pini* | MEAN 1092 | Portugal | MT374680 | MT374693 | MT374705 |
| *P. pinicola* | KUMCC 19-0183^T^ | China | MN412636 | MN417509 | MN417507 |
| *P. portugalica* | CBS 393.48^T^ | Portugal | KM199335 | KM199510 | KM199422 |
|  | LC4324 | China | KX895001 | KX895219 | KX895333 |
| *P. rhizophorae* | MFLUCC 17-0416^T^ | Thailand | MK764283 | MK764327 | MK764349 |
| *P. rhododendri* | IFRDCC 2399^T^ | China | KC537804 | KC537811 | KC537818 |
| *P. rhodomyrtus* | CFCC 54733 | China | OM746310 | OM840082 | OM839983 |
|  | CFCC 55052 | China | OM746311 | OM840083 | OM839984 |
| *P. rosea* | MFLUCC12-0258^T^ | China | JX399005 | JX399069 | JX399036 |
| *P. scoparia* | CBS 176.25^T^ | China | KM199330 | KM199478 | KM199393 |
| *P. sequoiae* | MFLUCC 13-0399^T^ | Italy | KX572339 | – | – |
| *P. shaanxiensis* | CFCC 54958^T^ | China | ON007026 | ON005043 | ON005054 |
|  | CFCC 57356 | China | ON007027 | ON005044 | ON005055 |
| *P. shorea* | MFLUCC12-0314^T^ | Thailand | KJ503811 | KJ503817 | KJ503814 |
| *P. silvicola* | CFCC 55296^T^ | China | ON007032 | ON005049 | ON005060 |
|  | CFCC 54915 | China | ON007033 | ON005050 | ON005061 |
|  | CFCC 57363 | China | ON007034 | ON005051 | ON005062 |
| 1. ***solicola*** | **SAUCC003804^T^** | **China** | **OQ692020** | **OQ718737** | **OQ718795** |
|  | **SAUCC003806** | **China** | **OQ692021** | **OQ718738** | **OQ718796** |
|  | **SAUCC003807** | **China** | **OQ692022** | **OQ718739** | **OQ718797** |
| *P. spatholobi* | SAUCC231201^T^ | China | OQ692023 | OQ718740 | OQ718798 |
|  | SAUCC231203 | China | OQ692024 | OQ718741 | OQ718799 |
|  | SAUCC231204 | China | OQ692025 | OQ718742 | OQ718800 |
|  | SAUCC231213 | China | OQ692026 | OQ718743 | OQ718801 |
| *P. spathulata* | CBS 356.86^T^ | Chile | KM199338 | KM199513 | KM199423 |
| *P. spathuliappendiculata* | CBS 144035^T^ | Australia | MH554172 | MH554607 | MH554845 |
| *P. telopeae* | CBS 114137 | Australia | KM199301 | KM199559 | KM199469 |
|  | CBS 114161^T^ | Australia | KM199296 | KM199500 | KM199403 |
|  | CBS 113606 | Australia | KM199295 | KM199498 | KM199402 |
| *P. terricola* | CBS 141.69^T^ | Pacific Islands | MH554004 | MH554438 | MH554680 |
| *P. thailandica* | MFLUCC 17-1617^T^ | Thailand | MK764285 | MK764329 | MK764351 |
| *P. trachycarpicola* | OP068^T^ | China | JQ845947 | JQ845946 | JQ845945 |
|  | IFRDCC 2403 | China | KC537809 | KC537816 | KC537823 |
|  | LC4523 | China | KX895011 | KX895230 | KX895344 |
| *P. unicolor* | MFLUCC12-0276^T^ | China | JX398999 | – | JX399030 |
|  | MFLUCC12-0275 | China | JX398998 | JX399063 | JX399029 |
| *P. verruculosa* | MFLUCC12-0274^T^ | China | JX398996 | JX399061 | – |
| *P. yanglingensis* | LC4553^T^ | China | KX895012 | KX895231 | KX895345 |
|  | LC3412 | China | KX894980 | KX895197 | KX895312 |
| *P. yunnanensis* | HMAS 96359^T^ | China | AY373375 | – | – |

Notes: Ex-type or ex-epitype strains are marked with “T” and the new species information described in this study is marked in bold.
